# Interleukin‐4 administration improves muscle function, adult myogenesis, and lifespan of colon carcinoma‐bearing mice

**DOI:** 10.1002/jcsm.12539

**Published:** 2020-02-27

**Authors:** Domiziana Costamagna, Robin Duelen, Fabio Penna, Detlef Neumann, Paola Costelli, Maurilio Sampaolesi

**Affiliations:** ^1^ Translational Cardiomyology, Stem Cell Biology and Embryology, Department of Development and Regeneration University Hospital Gasthuisberg Leuven Belgium; ^2^ Experimental Medicine and Clinical Pathology Unit, Department of Clinical and Biological Sciences University of Turin Turin Italy; ^3^ Institute of Pharmacology Hannover Medical School, MHH Hannover Germany; ^4^ Human Anatomy Unit, Department of Public Health, Experimental and Forensic Medicine University of Pavia Pavia Italy

**Keywords:** Cancer‐induced skeletal muscle atrophy, Interleukin‐4, Survival curve, Muscle function, Muscle regeneration, Protein synthesis

## Abstract

**Background:**

Anorexia, body wasting, inflammation, muscle, and adipose tissue loss are hallmarks of cancer cachexia, a syndrome that affects the majority of cancer patients, impairing their ability to endure chemotherapeutic therapies and reducing their lifespan. In the last 10 years, alterations of protein turnover and impairment of adult myogenesis have been proposed as major contributing factors.

**Methods:**

Muscle stem cells, including satellite cells, mesoangioblasts, and fibroadipogenic progenitors, were isolated and characterized from C26 colon carcinoma‐bearing (C26) mice. Circulating levels of interleukin‐4/13 (IL4/IL13) were analysed by ELISA, and the effects of IL4 on muscle mass and function, protein synthesis, muscle regeneration, and myogenic progenitor cell number were analysed at both functional (treadmill and grip test) and molecular levels (qRT–PCR, immunofluorescence analysis, surface sensing of translation, and western blot). The Kaplan–Meier test was used to analyse the survival curve of IL4‐treated and IL4‐untreated C26 mice.

**Results:**

The administration of IL4 to C26 mice rescued muscle mass by increasing protein synthesis. The IL4 treatment improved performances and prolonged survival of C26 mice. IL4 administration re‐established both number and function of satellite cells and fibroadipogenic progenitors without affecting mesoangioblasts in C26 mice, rescuing myogenesis. Upon IL4 treatment, a high number of cytotoxic lymphocytes and type II macrophages were observed with a subsequent increase in necrotic areas of C26 tumours.

**Conclusions:**

The results here presented shed new light on IL4 signalling during muscle wasting and early stages of muscle regeneration that explain the beneficial effect observed in IL4‐treated C26 mice. These findings might aid to develop therapeutic approaches to improve mobility and quality of life in cachectic patients.

## Introduction

Cachexia is a multifactorial syndrome associated to many types of cancers, affecting mainly gastrointestinal tract and lungs, that compromises absorption of nutrients and decreases the possibility for the patients to efficiently breathe.^1^ The occurrence of cachexia increases chemotherapy toxicity, complicates patient management, and enhances mortality rates, accounting for about 20% of cancer deaths worldwide (Cancer fact sheet, World Health Organization 2017; https://www.who.int/news‐room/fact‐sheets/detail/cancer).

Loss of muscle, with or without loss of fat mass, is one of the main hallmarks of the cachectic phenotype, is not reversed by nutrient supplementation, and, at least in experimental model systems, is mainly associated with the hyperactivation of catabolic signals involving the main proteolytic systems.^2^, ^3^, ^4^ In addition, protein synthesis is altered in favour of degradation and cannot be restored in experimental models by forcing the activity of anabolic pathways.^5^ Cytokines and pro‐inflammatory signals produced by the host immune system interact with the tumour, contributing to generate insulin and other growth factors resistance in the muscle of cancer patients. Consistently, drugs able to enhance muscle anabolism and to increase appetite and energy storage, such as several ghrelin mimetics, have entered in phase II clinical trials.^6^

In the last decade, different groups have proposed that also impaired myogenesis could contribute to cancer‐induced muscle wasting. In this regard, in the muscle of tumour‐bearing animals, satellite cells (SCs) increase in number and accumulate in the interstitial space among the fibres.^7^, ^8^ The same pattern has been observed also in cancer patients.^8^, ^9^ From the mechanistic point of view, activation of NF‐κB in myogenic progenitors has been pointed as responsible for a persistent up‐regulation of PAX7, thus blocking the expression of transcription factors important for SC differentiation.^8^ This myogenic impairment can be partially removed by treatment with MEK inhibitors.^7^ Another study has shown the occurrence of a defect in muscle stem cell proliferation and differentiation in cachectic animals, which has been associated with decreased number of macrophages and mesenchymal progenitors.^10^

Several studies have clarified that, in addition to SCs, also other cell types can contribute to myogenesis, such as mesoangioblasts (MABs), vessel‐associated muscle progenitors, and fibroadipogenic progenitors (FAPs). Consistently, recent data from our group have demonstrated that BMP‐SMAD signalling blockade improves MAB myogenic differentiation.^11^ As for FAPs, they have been firstly described as non‐myogenic interstitial stem cells present in the adult muscle that upon injury can drive SC differentiation through secretion of interleukin‐4 (IL4) and interleukin‐13 (IL13).^12^, ^13^ Indeed, these cells have been reported to improve skeletal muscle regeneration in acute^14^ and chronic conditions.^15^ On the other side, IL4/IL4R and IL13/IL13R have been found highly up‐regulated in the skeletal muscle after exercise, suggesting an anabolic role played by the two cytokines in this tissue.^16^ IL4 has also been shown to recruit myoblasts to form mature myotubes,^17^ and further studies have demonstrated that this cytokine can exert a pro‐migratory effect *in vitro*.^18^ Interestingly, in pancreatic cancer patients affected by cachexia, IL4 messengers are down‐regulated in liver and muscle.^19^

The aim of the present study is to investigate the mechanisms underlying the impaired myogenesis in cancer hosts, focusing on the role that interstitial cells and cytokines involved in muscle differentiation might play in contributing to cancer‐induced muscle wasting.

## Materials and methods

### Animal experiments

All animal procedures^20^ were performed according to the guidelines of the Animal Welfare Committee of KU Leuven and Belgian/European legislation (P051/2017). Four‐week‐old Balb/c mice of approximately 20 g were obtained from Charles River Laboratories (Wilmington, MA, USA). Mice were maintained on a regular dark–light cycle (light from 8 h a.m. to 8 h p.m.), with free access to food and water. C26 colon‐adenocarcinoma cells^5^ were maintained in high‐glucose Dulbecco's modified Eagle's medium (DMEM), supplemented with 10% foetal bovine serum (FBS), 1 mg/mL of sodium pyruvate, and 1 IU/mL of penicillin and streptomycin. The plates were incubated at 37°C, 5% CO_2_, 5% O_2_, and the medium was refreshed every 2 days. Tumour‐bearing mice were inoculated subcutaneously dorsally with 5 × 10^5^ C26 carcinoma cells. The number of mice per group depends on the experiments as it is indicated in the legends of the respective assays. Where indicated, 1.3 μg per mouse intraperitoneal IL4 (Peprotech, Rocky Hill, NJ, USA) was administered every day, starting from Day 5 to the day of sacrifice.^14^ Multiple experiments were performed with body weight, tumour size, and survival rates recorded. The blood was collected through intracardiac injection at the sacrifice, obtained through cervical dislocation. After collecting the blood in heparin, centrifugation at 845 g was performed to obtain the blood cells (fixed with Thermo Fisher Scientific, 1‐step Fix/Lyse Solution for flow cytometry analysis), and the plasma was kept at −80°C for ELISA experiments. Animal weight and food intake were recorded daily. Tumour‐bearing mice were sacrificed under anaesthesia 13–14 days after tumour transplantation. Several muscles and organs were rapidly excised, weighed, frozen in isopentane cooled with liquid nitrogen, and stored at −80°C. Peritoneal lavage was performed with 50 μM of EDTA in phosphate‐buffered saline (PBS) to extract lymphocytes and macrophages. Tumour tissue was digested for 90′ in 1 mg/mL collagenase II and IV 1:1.

Four‐month‐old alpha‐sarcoglycan (αSG)‐null mice from the internal colony of Sampaolesi laboratory were generated by the group of K.P. Campbell (University of Iowa, IA, USA).^21^ Due to the absence of αSG from the muscle membrane, these mice, starting from 2 to 3 months of age, undergo continuous cycle of muscle fibre degeneration and regeneration. For these reasons, these mice were used to test the ability of C‐MABs and C26‐MABs to contribute to muscle regeneration upon cyclosporine immunosuppression (as already described in Costamagna *et al*.^11^).

### Survival experiment

Mice were followed until the KU Leuven predetermined humane endpoint criteria were fulfilled. Mice were ultimately sacrificed when C26 cell inoculation determined body mass loss above 20%, combined with an overall health status of severe sufferance, food intake decrease, abnormal appearance and posture (lack of grooming, piloerection, and hunched posture), unnatural behaviour (impaired locomotion, inactivity, and reduced reactivity to external stimuli), and body temperature below 28°C. During the experiment, mice were separated to avoid self‐mutilation.

### Treadmill and muscle strength

Mice were introduced to the treadmill belt to test the conditions before the actual test (motor speed set to zero, for 5 min). Later on, the motor speed was set to 10 m/min, with a 1 m/min increase and an uphill inclination of 10°, till exhaustion and >10 s stop.^11^ Grip strength was measured with digital grip strength metre (Columbus Instruments). Three measurements with 10 min interval between tests were completed for each mouse.

### Muscle injury

Mice received 50 μL injection of 10 μM solution of cardiotoxin (CTX, *Naja mossambica mossambica*, Sigma‐Aldrich) in the tibialis anterior, the gastrocnemius, and the quadriceps of one hind limb. The contralateral muscles were injected with the same volume of PBS as a control. Ten days after the *in vivo* regeneration experiments, the mice were sacrificed by cervical dislocation, and muscles were processed for histological analysis. For the experiment of muscle injury in parallel to the survival experiment (regenerating muscle shown in Supporting Information, *Figure S6b*, *n* = 5 C26; *n* = 5 C26 + IL4), mice were sacrificed when all the signs of severe sufferance were reached and body temperature was below 28°C.

### Surface Sensing of Translation (SUnSET) experiment

In order to detect protein synthesis at the moment of the sacrifice, mice were administered intraperitoneally of 0.040 μmol/g puromycin (Sigma‐Aldrich) and euthanized 30 min after injection.^22^ Muscles were rapidly removed, weighted, and frozen in liquid nitrogen. Puromycin‐conjugated peptides were visualized by western blotting (WB) on a gradient gel by using a mouse monoclonal anti‐puromycin antibody (*Table* 2). The density of each lane has been then measured by QuantityOne software and normalized per aTubulin (*Table* 2) levels to determine the protein synthesis rate expressed as puromycin relative abundance.

### Cell isolation and culture

Adult SCs were isolated by enzymatic digestion with 0.01% collagenase D and 0.06% pancreatine during 90′ digestion of minced murine muscle and pre‐plated on 0.1% gelatin‐coated dishes to remove fibroblasts. Cells were counted and plated on 0.1% collagen for experiments of cell proliferation in high‐glucose DMEM supplemented with 20% FBS, 1 mg/mL of sodium pyruvate, 1 IU/mL of penicillin and streptomycin, and 1 mg/mL of non‐essential amino acids, 50 μg/mL of gentamicin, and 1:100 chick embryo extract (Gentaur, Genprice Inc., CA, USA). The plates were incubated at 37°C, 5% CO_2_, 5% O_2_, and the medium was refreshed every 2 days. Myoblast differentiation was obtained with the same medium as for C2C12 differentiation. After tissue explant, adult Mesoangioblasts (MABs) were sorted for ALP+ and induced to express GFP (as for 11). After sorting, the GFP^+^ cells were cultured on 0.1% collagen‐coated dishes and maintained in high‐glucose DMEM supplemented with 20% FBS, 1 mg/mL of sodium pyruvate, 1 IU/mL of penicillin and streptomycin, and 1 mg/mL of non‐essential amino acids. The plates were incubated at 37°C, 5% CO_2_, 5% O_2_, and the medium was refreshed every 2 days. Intramuscular injections of 2 × 10^5^ murine GFP^+^ MAB or MAB cells pretreated for 48 h with 5 μM dorsomorphin (Sigma‐Aldrich; DM‐MABs) were performed in αSG‐null mice (*n* = 5 for each group). During the entire treatment, mice were maintained with cyclosporine (Sandimmune cyclosporine, Novartis; 10 mg/kg); 21 days post‐injection mice were sacrificed and compared with sham‐operated mice (saline solution injected).

C2C12 myoblast cells were maintained at 37°C in 5% CO_2_ in air‐humidified chamber in high‐glucose DMEM with glutamine, supplemented with 10% FBS, 1 mg/mL of sodium pyruvate, and 1% penicillin/streptomycin (Thermo Fisher Scientific). When the cells reached confluency, growth medium was replaced with differentiation medium, supplementing high‐glucose DMEM with 2% horse serum, 1 mg/mL of sodium pyruvate, and 1 IU/mL of penicillin/streptomycin. The plates were incubated at 37°C, 5% CO_2_, 5% O_2_, and the medium was refreshed every 2 days.

In order to efficiently knockdown *IL4Ra* and *IL13Ra*, endoribonuclease‐prepared siRNAs against *IL4Ra* and *IL13Ra* (MISSION esiRNA Mouse *Il4ra* and MISSION esiRNA Human *IL13ra1*; Sigma‐Aldrich) were applied following the manufacturer's instructions. Briefly, C2C12 at Day 2 of myotube differentiation was transfected using Lipofectamine 2000 and Opti‐MEM. At different time points after transfection (24, 48, and 72 h), cells were lysed for RNA extraction or WB analysis. RNA Mini Kit was used for RNA isolation, and genomic DNA traces were removed with Turbo DNase, following the manufacturer's instructions; 500 μg of RNA were reverse‐transcribed with the SuperScript III kit. The resulting cDNA was transferred in a 384‐well plate pre‐filled with 250 nM primers. Platinum SYBER green mix was added to a final volume of 10 μL per well. The RT–qPCR was performed for 40 cycles (95°C, 15 s; 60°C, 45 s) and read on a ViiA 7 qPCR plate reader. The list of murine primers used is available in *Table* 1.

**Table 1:** List of the primers used in this study

IL13 fw	CAGCCTCCCCGATACCAAAAT
IL13 rev	GCGAAACAGTTGCTTTGTGTAG
IL13R fw	ATGCTGGGAAAATTAGGCCATC
IL13R rev	ATTCTGGCATTTGTCCTCTTCAA
IL4 MC13 fw	GGTCTCAACCCCCAGCTAGT
IL4 MC13 rev	CCCTTCTCCTGTGACCTCGT
IL4R fw	GAATAGGCCGGTCCAATCAGA
IL4R rev	CAGCCATTCGTCGGACACATT
Myomixer fw	CTGAGCTGTCTGCTCTTTGT
Myomixer rev	TCTCCTTCCTCTGGGAGTG
Myomaker fw	CCTGCTGTCTCTCCCAAG
Myomaker rev	AGAACCAGTGGGTCCCTAA
Myomerger‐short fw	CAGGAGGGCAAGAAGTTCAG
Myomerger‐short rev	ATGTCTTGGGAGCTCAGTCG
Myomerger‐long fw	ACCAGCTTTCATGCCAGAAG
Myomerger‐long rev	ATGTCTTGGGAGCTCAGTCG
Gapdh fw	TGGTGAAGGTCGGTGTGAAC
Gapdh rev	GCTCCTGGAAGATGGTGATGG
Tbp fw	CAAACCCAGAATTGTTCTCCTT
Tbp rev	ATGTGGTCTTCCTGAATCCCT
Hprt fw	TGGATACAGGCCAGACTTTGTT
Hprt rev	CAGATTCAACTTGCGCTCATC

### Flow cytometry analysis

Staining was performed with primary antibody (*Table* 2) during 30′ at room temperature, and it was followed by 30′ secondary antibody staining (just for Ki67, Alexa Fluor 647, Thermo Fisher Scientific) or from final washes. Fluorescence minus one and compensations were included when necessary for appropriate gating. Cells were analysed and quantified by flow cytometry (BD FACS Canto HTS; BD Biosciences), and FlowJo Software was used to analyse the data (FlowJo V10 LLC).

**Table 2:** List of the antibodies used in this study for western blot (WB), immunofluorescence (IF), fluorescence‐activated cell sorting (FACS) and Flow Cytometry analyses

Antibody	Provider	WB	IF	FACs
PE Ms Human/Mouse/Rat Alkaline Phosphatase MAb (Cl B4‐78)	R&D Systems			2.5 μg/10^6^
FITC rat anti‐mouse CD11b	BD Pharmigen			1 μg/mL
APC F4/80 Monoclonal (BM8)	Thermo Fisher Scientific			1 μg/mL
Brilliant Violet 421 anti‐mouse CD206 (C068C2)	BioLegend			1 μg/mL
APC anti‐mouse CD3	BioLegend			1 μg/mL
APC/Cy7 anti‐mouse CD4 (Cl GK1.5)	BioLegend			1 μg/mL
PerCP/Cy5,5 anti‐mouse CD8a	BioLegend			1 μg/mL
PE‐anti‐mouse CD25	BioLegend			1 μg/mL
APC Ly‐6A/E (Sca‐1) Monoclonal (D7)	Thermo Fisher Scientific			1 μg/mL
PE‐Vio770‐Anti‐mouse Integrin a7 (Cl 3C12)	Miltenyi Biotech			1 μg/mL
Ms anti‐human/Mouse/Rat Ki67 (Cl B56)	BD Pharmigen		1:300	1 μg/mL
Goat GFP (ab5450)	Abcam		1:500	
Rabbit GFP	Thermo Fisher Scientific		1:300	
Mouse Human/Mouse/Rat MF20	DSHB	1:3	1:10	
Mouse Human/Mouse/Rat eMyHC	DSHB	1:3	1:10	
Mouse Human/Mouse/Rat PAX7	DSHB	1:3	1:10	
Mouse Human/Mouse/Rat Myogenin (DH5)	DSHB	1:3	1:10	
Mouse PDGF R alpha	R&D Systems		1:100	
Anti‐Mannose Receptor (15‐2) (ab8918)	Abcam	1:200	1:100	
Anti‐F4/80 [CI:A3‐1] (ab6640)	Abcam	1:100	1:50	
Rabbit Mouse P‐Akt	Cell Signaling	1:500		
Rabbit Mouse Akt	Cell Signaling	1:1000		
Rabbit P‐p70	Cell Signaling	1:1000		
Mouse p70	Cell Signaling	1:1000		
Rabbit JNK	Cell Signaling	1:1000		
Rabbit P‐p38	Cell Signaling	1:1000		
Mouse p38	Santa Cruz Biotech	1:200		
Mouse p‐ERK	Santa Cruz Biotech	1:200		
Rabbit ERK	Santa Cruz Biotech	1:200		
Mouse ESGP Antibody AF4580	R&D Systems	1:3000	1:1000	
Rabbit IL4Ra (SAB4501541)	Sigma‐Aldrich	1:200		
Rabbit TRIM63 Antibody (SAB1411517)	Sigma‐Aldrich	1:500		
Rabbit Beclin1‐BH3 Domain (SAB1306537)	Sigma‐Aldrich	1:1000		
Rabbit LC3B Antibody (L7543)	Sigma‐Aldrich	1:1000		
Mouse p62	BD Biosciences	1:10 000		
Atrogin1 (AP 2041)	ECM Biosciences	1:200		
Mouse Human/Mouse/Rat aTubulin	Sigma‐Aldrich	1:1000		
Rabbit Human/Mouse/Rat GAPDH	Sigma‐Aldrich	1:1000		
Mouse Puromycin MABE343 (Cl 12D10)	Merk Millipore	1:1000		

### ELISA assay

IL4 and IL13 serum levels were detected by a commercially available mouse ABTS‐ELISA kit, used according to the manufacturer's instructions (Peprotech). Serum from each animal (50 μL) was assayed in triplicate. Quantitative calibration was obtained performing a standard curve with recombinant mouse IL4 and IL13.

### Immunofluorescence

Tissues (7 μm muscle tissue and 5 μm tumour tissue) and cells were fixed with 4% paraformaldehyde (Polysciences Europe GmbH) in PBS and permeabilized with 0.2% Triton X‐100 in PBS containing 1% (w/v) bovine serum albumin (BSA). A blocking solution containing donkey serum (1:10 diluted in PBS; VWR) was applied, and primary antibodies (*Table* 2) were incubated overnight in PBS supplemented with 1% BSA. Secondary Alexa Fluor donkey antibodies were diluted at 4 μg/mL in PBS supplemented with 1% BSA, and the nuclei were counterstained with 10 μg/mL Hoechst. Fluor Save reagent (Millipore, Chemicon) was used as mounting medium. Images were acquired using an Eclipse Ti inverted microscope (Nikon).

### Western blot

Western blotting analysis was performed on lysates from tissue or cells in RIPA buffer (Sigma‐Aldrich) supplemented with 10 mM sodium fluoride, 0.5 mM sodium orthovanadate, 1:100 protease inhibitor cocktail (Sigma‐Aldrich), and 1 mM phenylmethylsulfonyl fluoride. Equal amounts of protein (40 μg) were heat‐denatured in sample‐loading buffer (50 mM Tris–HCl, pH 6.8, 100 mM DTT, 2% SDS, 0.1% bromophenol blue, 10% glycerol), resolved by SDS‐polyacrylamide gel electrophoresis, and then transferred to nitrocellulose membranes (Protran, nitrocellulose membrane, Amersham). The filters were blocked with Tris‐buffered saline containing 0.05% Tween and 5% non‐fat dry milk (Sigma‐Aldrich) and then incubated overnight with the indicated dilutions of different primary antibodies (*Table* 2). All secondary horseradish peroxidase‐conjugated antibodies (BioRad) were diluted 1:5000 in Tris‐buffered saline‐Tween and 2.5% non‐fat dry milk (Sigma‐Aldrich). After incubation with SuperSignal Pico or Femto chemiluminescence substrate (Thermo Scientific), the polypeptide bands were detected with GelDoc chemiluminescence detection system (BioRad). Quantification of relative densitometry was obtained by normalizing vs. background and housekeeping proteins using the QuantityOne software (BioRad).

### Statistical analysis

All results were expressed as mean ± standard deviation, with the exception of gene expression (mean ± SEM). Representative WBs were selected from independent experiments. When data distribution approximated normality and two groups were compared, a Student's *t*‐test was used. When three or more groups were compared, a one‐way analysis of variance was used. In case of non‐normal distribution of the data pools, such as for statistical comparison of the differences between percentage values, a Kruskal–Wallis one‐way analysis of variance by rank test was used. All statistical tests were performed via Prism software (GraphPad).

## Results

**Figure 1:**
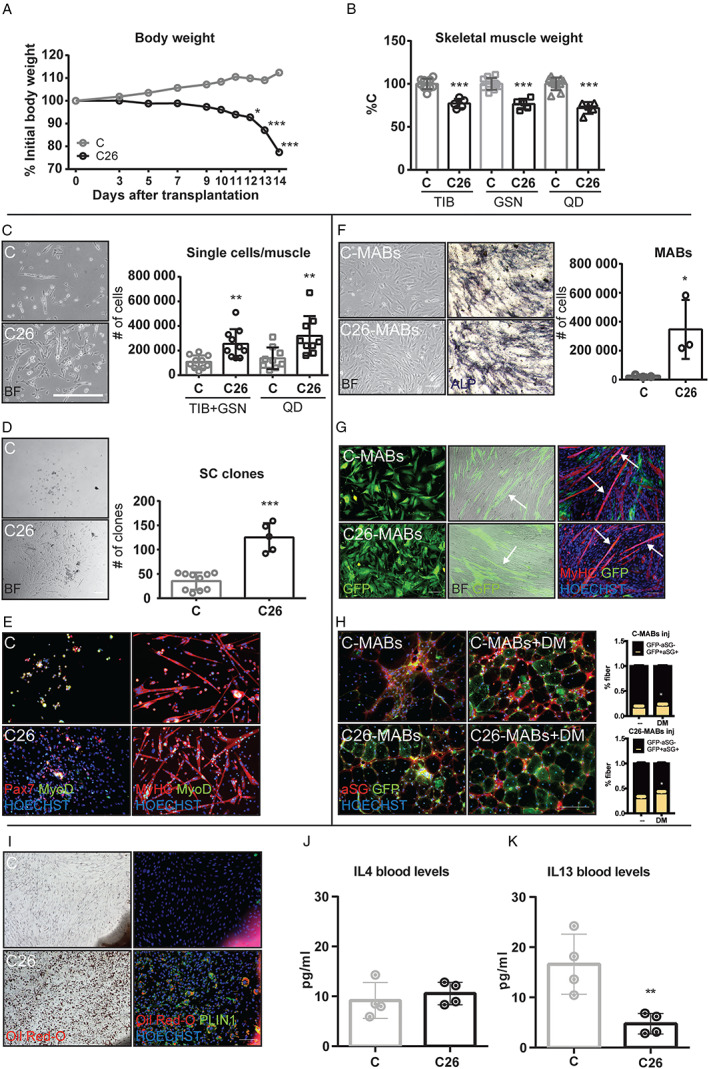
Characterization of control (C) and C26 muscle stem cells. (*A*) Body weight loss of C (*n* = 6) and C26 mice (*n* = 8) during 14 days of tumour growth, expressed as percentage of initial body weight. (*B*) *Tibialis anterior* (TIB; C: 98 ± 5.9 mg/100 g i.b.w.), *gastrocnemius* (GSN; C: 280.5 ± 19 mg/100 g i.b.w.), and *quadriceps* (QD; C: 396 ± 29 mg/100 g i.b.w.) muscle weight from C and C26, expressed as percentage of the initial body weight. (*C*) BF images (on the left) and quantification (on the right) of satellite cells (SCs) extracted from C‐specific and C26‐specific muscles. (*D*) Representative SCs clones (on the left) and quantification (on the right) from C and C26, 4 days after seeding. (*E*) IF of SCs from C and C26 stained for PAX7 (red) and MyoD (green; column on the left = Day 0 of differentiation), or MyHC (red) and MyoD (green; column on the right = Day 2 of differentiation). (*F*) BF images of MABs isolated (column on the left) from C and C26 muscle explants after 7 days of organ culture and stained for ALP (column on the right). Quantification of C‐MABs and C26‐MABs (graph on the right). (*G*) GFP^+^ C‐MABs and C26‐MABs (column on the left), BF overlapped with fluorescent images of the same cells with C2C12 in co‐culture (middle column). IF for MyHC (red) and GFP (green) on GFP^+^ C‐MABs and C26‐MABs in co‐culture with C2C12 after 5 days of differentiation medium (column on the right). (*H*) Transversal sections of *TIB* from αSG‐null mice injected with GFP^+^C‐MABs or GFP^+^C26‐MABs (column on the right) or with the same cells pretreated during 48 h with dorsomorphin (5 μM, DM), before the injection. Quantification (on the right) of the percentage of GFP^+^ αSG^+^ fibres in the injected muscles. (*I*) C and C26 populations of adipocyte‐like cells were stained with Oil‐Red‐O (column on the left) and perilipin (PLIN1; column on the right). Nuclei were stained with HOECHST (blue). Plasma levels for (*J*) IL4 (pg/mL) and (*K*) IL13 (pg/mL) cytokines in C and C26 mice. Significance of the differences: ^*^ *P* < 0.05, ^**^ *P* < 0.01, ^***^ *P* < 0.001 vs. C. Scale bar: 500 μm.

Mice bearing the C26 tumour represent a well‐characterized system to study cancer cachexia.^5^ As previously reported, 14 days after C26 cell implantation, body weight in tumour‐bearing mice was about 75% of that observed in control animals (C; body weight at Day 0 = 20.41 ± 1.89 g; *Figure* 1A). A comparable reduction (about 25% lower than C values) was observed for several muscles, namely, *tibialis anterior*, *gastrocnemius*, and *quadriceps* (*Figure* 1B).

### Muscle stem cells isolated from C26 hosts: *in vitro* proliferation and differentiation

The number of SCs isolated from muscles of C26 hosts (hereafter C26‐SCs) was higher than that obtained from C muscles (C‐SCs; *Figure* 1C). Consistently, 4 days after plating, C26‐SC cultures displayed a higher number of clones than C‐SC ones (*Figure* 1D), demonstrating that the proliferative ability was higher in C26‐SCs than in C‐SCs. In immunofluorescence analysis, both C26‐SCs and C‐SCs showed positivity for PAX7 and MyoD, confirming *in vitro* their myogenic lineage (*Figure* 1E, left panels). When exposed to differentiation medium, these cells were able to activate the differentiation programme, fusing into multinucleated myotubes and expressing both MyoD and MyHC (*Figure* 1E, right panels), or ACTN2 (Supporting Information, *Figure* *S1* *a*). The expression of terminally differentiated myotube markers let us conclude that *ex vivo* C26‐SCs behave comparably with C‐SCs.

Similarly, MABs sorted as alkaline phosphatase (ALP)‐positive cells (*Figure* 1F) were isolated from the skeletal muscle of C26 hosts in higher number than in C mice (*Figure* 1F, quantification). To evaluate the ability of MABs to contribute to muscle differentiation, C‐MABs and C26‐MABs were transfected to express GFP (*Figure* 1G, left panels) and co‐cultured with C2C12 myoblasts. After 5 days of differentiation, GFP‐expressing multinucleated myotube‐like structures appeared (*Figure* 1G, central panels). MyHC^+^ and GFP^+^ myotubes were detected in both C‐MABs and C26‐MABs co‐cultures, demonstrating that GFP^+^MABs were able to fuse with C2C12 myotubes (*Figure* 1G, right panels; Supporting Information, *Figure* *S1* *b* and *S1* *c*). In order to test if C‐MABs and C26‐MABs display the same ability *in vivo*, these cells were injected intramuscularly into αSG‐null mice.^11^ Twenty‐one days after C‐MAB and C26‐MAB injection, the muscle of αSG‐null mice showed the presence of GFP^+^ fibres expressing αSG, clearly originated from the fusion of the injected C‐MABs and C26‐MABs (*Figure* 1H, left panels). Moreover, as previously described,^11^ both engraftment and differentiation were further improved when injected C‐MABs and C26‐MABs were pretreated in culture for 48 h with 5 μM dorsomorphin (DM), in order to inhibit type I BMP receptor complex and obtain a proliferation arrest, finally promoting differentiation (*Figure* 1H, right panels and quantification). Taken together, these results confirm that the myogenic properties of MABs isolated from C26 hosts are not impaired.

Finally, the majority of interstitial muscle cells, isolated from the muscle of C26‐bearing mice and put in culture, differentiated to adipocyte‐like cells, a pattern that was not observed in cultures prepared with cells isolated from C muscles. Increased PLIN1 expression of Oil‐Red‐O‐positive cells (*Figure* 1I, right panel) and the positive bright field staining for Oil‐Red‐O (*Figure* 1I, left) confirmed the acquisition of an adipocyte‐like phenotype *in vitro*.

These data demonstrate that the impaired myogenesis occurring in the C26 hosts does not result from a stem cell autonomous defect but is rather related to the micro‐environment these cells are exposed to. The hypothesis is that tumour‐induced modulations of muscle micro‐environment do not allow stem cell differentiation. On the basis of the adipogenic drift observed when cells isolated from the muscle of C26 hosts were put in culture, the possibility that myogenic relevant cytokines^14^ could be perturbed was assessed. Data available in literature have stressed the importance of IL4 and IL13 during the first phases of muscle regeneration; thus, we evaluated their expression in both muscle and blood from C and C26 hosts. Quantitative qRT–PCR showed in muscle tissues from C26‐bearing mice a significant increase of IL4Ra and a decrease in IL13 transcript levels, with respect to control values, while no significant differences could be observed for both IL4 and IL13R mRNAs (Supporting Information, *Figure* *S1* *d*). The low availability of efficient antibodies for WB let however any conclusion on the expression of these molecules still at a speculative level. Finally, while circulating IL4 was comparable in both C26 hosts and C mice (*Figure* 1J), IL13 plasma levels were significantly lower in the former than in the latter (*Figure* 1K).

### Interleukin‐4 treatment improves cachexia in tumour‐bearing mice

**Figure 2:**
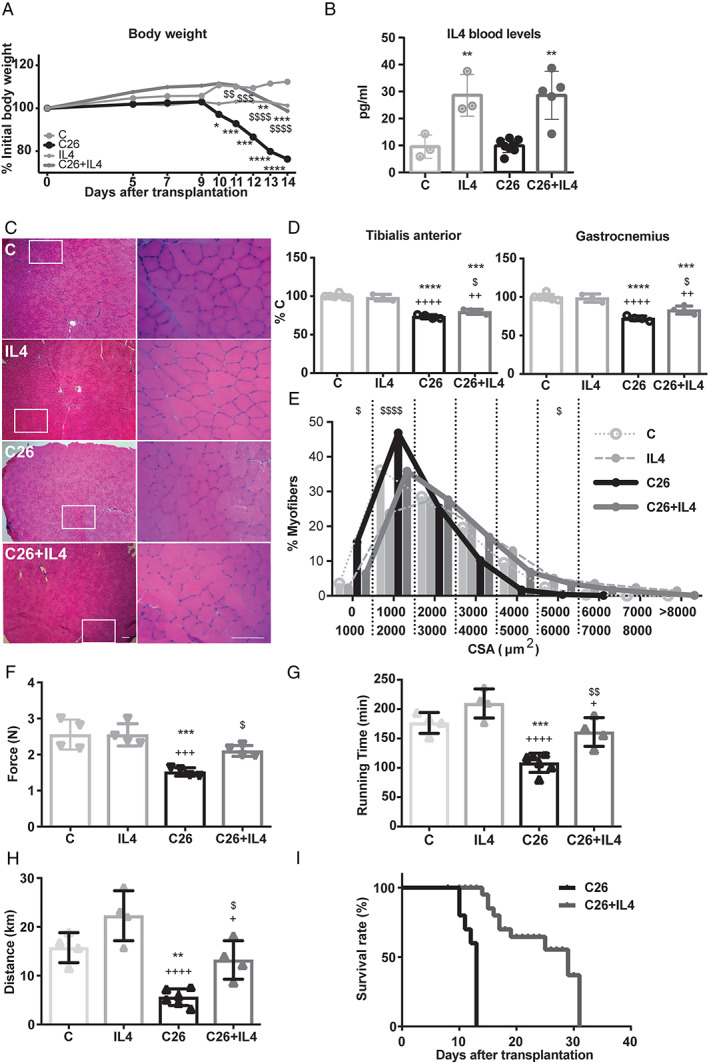
IL4 treatment counteracts muscle weight loss and fibre CSA, restores muscle performances, and prolongs survival of tumour‐bearing mice. (*A*) Percentage of body weight during 14 days after tumour injection in C26 (*n* = 6) and IL4‐treated tumour‐bearing mice (C26 + IL4, *n* = 6) was compared with control (C, *n* = 4) and IL4‐treated (IL4, *n* = 4) mice. (*B*) Plasma levels of IL4 (pg/mL) from C, C26, and daily treated IL4 and C26 + IL4 mice. (*C*) H&E staining (column on the left) of TIB muscles from the different groups with insets at a higher magnification (column on the right). (*D*) TIB (C: 105 ± 4 mg/100 g i.b.w.), GSN (C: 269 ± 7 mg/100 g i.b.w.), expressed as percentage of the initial body weight. (*E*) Fibre size distribution of *TIB* muscles. (*F*) Grip test analysis, (*G*) running time, and (*H*) distance measured by treadmill exhaustion assay for C26 and C26 + IL4 and their controls group, C and IL4. (*I*) Kaplan–Meier test for the significance of the difference between survival curve from C26 and C26 + IL4 attributed a *P* = 0.001 (*n* = 10 per group). Significance of the differences: ^*^ *P* < 0.05, ^**^ *P* < 0.01, ^***^ *P* < 0.001, ^****^ *P* < 0.0001 vs. C; ^+^ *P* < 0.05, ^++^ *P* < 0.01, ^+++^ *P* < 0.001, ^++++^ *P* < 0.0001 vs. IL4; ^$^ *P* < 0.05, ^$$^ *P* < 0.01, ^$$$^ *P* < 0.001, ^$$$$^ *P* < 0.0001 vs. C26.

The data reported earlier suggested that IL4/IL13 signalling was affected in the C26 hosts. The next step of the study was to investigate if potentiating such signalling could improve impaired regeneration and cachexia in the C26 hosts. The choice to use IL4 instead of IL13, as it could be expected looking at their circulating levels in the C26 hosts, was based on the observation that IL4 exerted its bioactivity engaging both its own receptor and the IL13 receptor (reviewed in Wu *et al*.^23^, thus providing a rather complete spectrum of action on the signalling pathways depending on both cytokines. Along this line, C26‐bearing mice were treated with IL4 after having excluded, by *in vitro* experiments, the possibility that IL4 could exert a direct effect on tumour cells (Supporting Information, *Figure S2a*). Starting from Day 5 after C26 inoculation, IL4 was administered intraperitoneally daily. IL4 absorption curve in treated control mice confirmed high circulating levels of the cytokine during the first 3 h after administration, followed by a reduction to control values, approximately 8 h after IL4 injection (Supporting Information, *Figure S2b*). Consistently, after 10 days of IL4 daily treatment, circulating levels of the cytokine were significantly increased in both C and C26 hosts (*Figure* 2B). To rule out the possibility of a local response of the immune system to IL4 administration, intraperitoneal lavage and flow cytometry analysis were performed. The results obtained showed, differently from what observed in positive controls (LPS‐injected mice; Supporting Information, *Figure S2e* and *S2h*), comparable lymphocyte (Supporting Information, *Figure S2f* and *S2g*) and macrophage (Supporting Information, *Figure S2i* and *S2j*) percentages and characteristics in IL4‐treated and IL4‐untreated C26 bearers. No differences were as well reported comparing the peritoneal lymphocytes and macrophages of IL4‐treated mice, confirming no effect at a peritoneal level after IL4 treatment (data not shown).

As expected, body weight in the C26 hosts was lower than in C (Day 0: 21.57 ± 1.07 g; 25% reduction), while it remained comparable with Day 0 in tumour‐bearing mice receiving IL4 (*Figure* 2A). In contrast, the reduction of food intake occurring in the C26‐bearing mice was not affected by IL4 treatment (mouse food intake on Day 13: C = 4.33 g; IL4 = 4.65 g; C26 = 1.45 g; C26 + IL4 = 1.88 g). Similarly, epididymal fat pad was completely depleted in both C26 and C26 + IL4 groups with respect to controls (C = 2.01 ± 0.57; IL4 = 2.63 ± 0.78 mg/100 g i.b.w.; C26 = ND; C26 + IL4 = ND; data are expressed as mg/100 g i.b.w., *P* < 0.22 C vs. IL4). IL4 treatment improved C26‐induced muscle wasting in terms of both muscle mass and cross‐sectional area (CSA) stabilizing this latter to the level of C (*Figure* 2C–2E and Supporting Information, *Figure S2d*). Because improved muscle mass or myofiber CSA do not necessarily mean better muscle function, an additional experiment was performed to obtain functional data. The results showed that grip strength was higher in IL4‐treated than in IL4‐untreated C26 hosts reaching values comparable with C mice (*Figure* 2F). Such improvement was confirmed by an exhaustion treadmill assay, showing that C26 hosts receiving IL4 run for longer time and distance than untreated C26 mice, similarly to C mice (*Figure* 2G and 2H).

Finally, a survival experiment demonstrated that IL4‐treated C26‐bearing mice were able to survive longer than untreated tumour hosts (31 days for C26 + IL4 vs. 13 days for C26; *Figure* 2I).

### Interleukin‐4 treatment increases intratumour cytotoxic lymphocytes and macrophages

**Figure 3:**
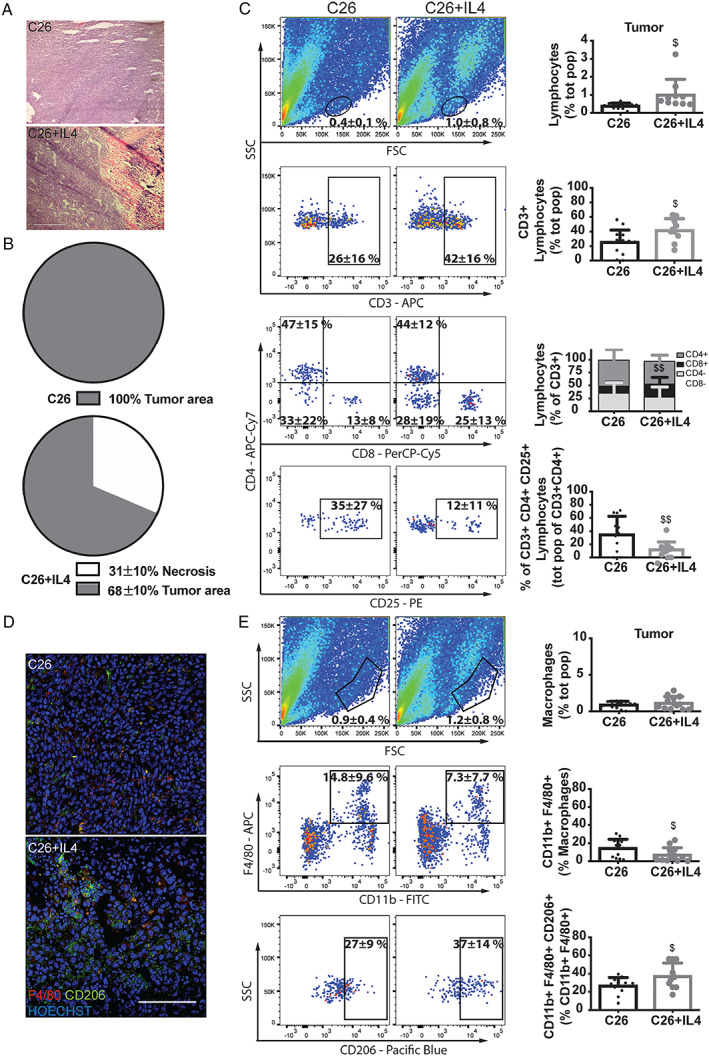
Tumour mass and flow cytometric analysis of tumour‐associated immune cells. (*A*) H&E staining, (*B*) quantifications of the necrotic area (eosin‐positive area—white) on the cell‐dense area (*haematoxylin‐*positive area—grey) of tumour masses from C26 and C26 + IL4 mice (*P* < 0.01 vs. C26). (*C*) Representative flow cytometry analysis and quantifications of the tumour digested cells from C26 and C26 + IL4 mice for the main markers of lymphocytes. (*D*) IF analysis of F4/80 and CD206 markers and (*E*) flow cytometry analysis of the tumour digested cells from C26 and C26 + IL4 mice for the same macrophage markers and quantifications. Significance of the differences: ^$^ *P* < 0.05, ^$$^ *P* < 0.01 vs. C26.

The administration of IL4 to the C26 hosts resulted in increased tumour mass (Supporting Information, *Figure S2c*) that however does not necessarily reflect an increased number of tumour cells. Indeed, the H&E staining revealed that more than 30% of tumour area in the IL4‐treated C26‐bearing mice (sacrificed at Day 31 after C26 cell injection) was necrotic (eosin positive) probably surrounded by small inflammatory cell infiltrate, a pattern that could not be observed in tumours isolated from C26 hosts (Day 13 after C26 implantation; *Figure* 3A and 3B).^24^ A possible limitation of these results is that the histological analysis compares Day 13 C26 with Day 31 C26 + IL4 tumours. However, because in the absence of IL4 treatment the C26 hosts die at Day 13 after tumour implantation, it was not possible to have untreated tumours at Day 31. Future investigation will assess if the peculiar histological aspect of C26 + IL4 tumours at Day 31 is already evident at Day 13.

IL4 treatment could contribute to stimulate the immune system against tumour cells. To investigate this point, C26 tumours from both IL4‐treated (Day 31 after tumour transplantation) and IL4‐untreated (Day 13 after tumour transplantation) animals were analysed in terms of lymphocyte and macrophage content. The results showed that the percentage of cytotoxic lymphocytes (CD3^+^CD8^+^) and type II macrophages (CD11b^+^, F4/80^+^, CD206^+^) was higher in tumours from IL4‐treated than IL4‐untreated C26 hosts (cytotoxic lymphocytes: C26 = 13 ± 8%, C26 + IL4 = 25 ± 13%, *Figure* 3C; macrophages: C26 = 27 ± 9%, C26 + IL4 = 37 ± 14%, *Figure* 3E). Consistently, the immunofluorescence indicated that the borders of necrotic areas in tumours from IL4‐treated C26 hosts were as well positive for F4/80 and CD206 (*Figure* 3D). Finally, the same analysis in the blood (Supporting Information, *Figure S3a* and *S3b*) revealed a decreased percentage of circulating T‐helper lymphocytes (CD3^+^CD4^+^) in IL4‐treated hosts.

### Interleukin‐4 administration improves protein synthesis in tumour‐bearing mice

**Figure 4:**
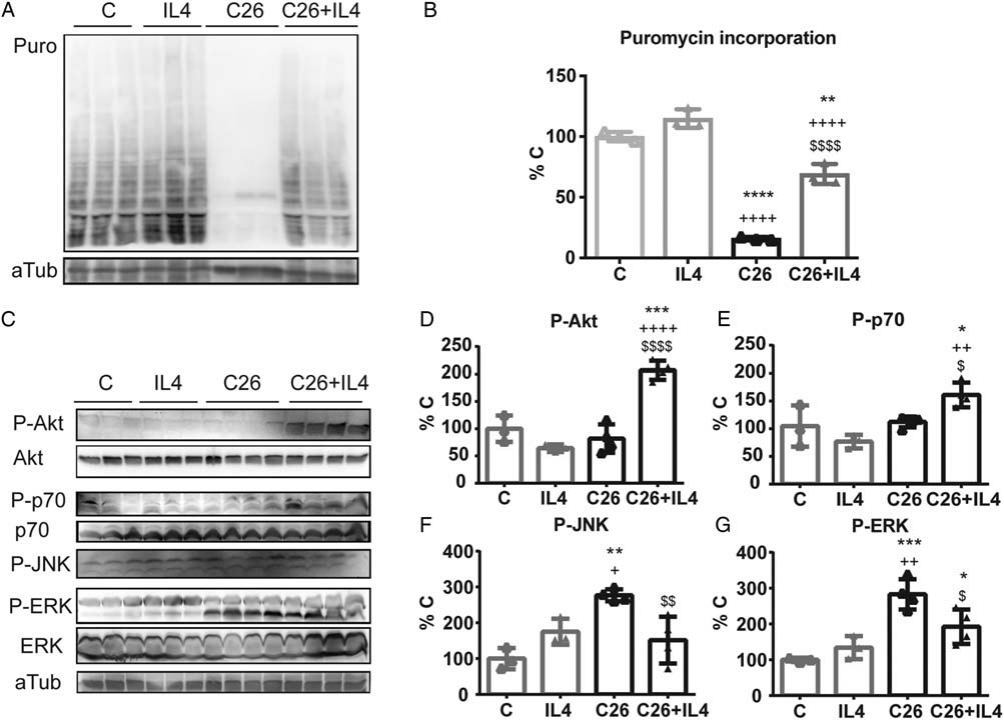
IL4 treatment is associated to rescue of muscle synthesis. (*A*) WB for puromycin (Puro) incorporation in newly synthetized muscle proteins from C, IL4, C26, and C26 + IL4 muscles was normalized on the levels of tubulin (aTub) levels and (*B*) quantified. (*C*) WB and (*D*) quantifications of P‐AKT, (*E*) P‐p70, (*F*) P‐JNK, and (*G*) P‐ERK levels normalized for the total protein levels and on aTub expression levels. Significance of the differences is reported as ^*^ *P* < 0.05, ^**^ *P* < 0.01, ^***^ *P* < 0.001 vs. C, ^****^ *P* < 0.0001; ^+^ *P* < 0.05, ^++^ *P* < 0.01, ^++++^ *P* < 0.0001 vs. IL4; ^$^ *P* < 0.05, ^$$^ *P* < 0.01, ^$$$$^ *P* < 0.0001 vs. C26.

The increased muscle mass and myofiber CSA could result from both reduced protein degradation and increased protein synthesis rates. An experiment was performed to assess this latter, by treating the animals with puromycin 30 min before sacrifice.^22^ The results showed that muscle protein synthesis is significantly improved in IL4‐treated than in IL4‐untreated C26 hosts (*Figure* 4A and 4B). Such improvement was associated with enhanced phosphorylation of Akt and p70, as shown by WB analysis (*Figure* 4C–4E). Moreover, the levels of P‐JNK and P‐ERK, this latter previously shown to correlate with muscle wasting in C26 hosts,^6^ decreased in the muscle of IL4‐treated C26‐bearing mice (*Figure* 4C, 4F, and 4G). On the other side, no significant differences among treated and untreated C26 hosts could be observed as for P‐p38 levels (Supporting Information, *Figure S4a* and *S4b*) and the state of activation of STAT3 and STAT6, both transcription factors reported to transduce the signals dependent on IL4Ra^25^, ^26^ (Supporting Information, *Figure S4c*–*S4e*). In addition, the main markers of proteolysis, such as Atrogin 1 and Trim 63 for the ubiquitin proteasome system (Supporting Information, *Figure S4f*–*S4h*) and Beclin1 and LC3B for the autophagic system (Supporting Information, *Figure S4i*–*S4l*), were not affected by IL4 treatment. Differently, the levels of p62‐SQSM1 were lower in IL4‐treated C26 mice when compared with the untreated ones and similar to controls (Supporting Information, *Figure S4i* and *S4l*–*S4n*).

### Interleukin‐4 treatment impinges on satellite cell accumulation in the muscle of C26 hosts

**Figure 5:**
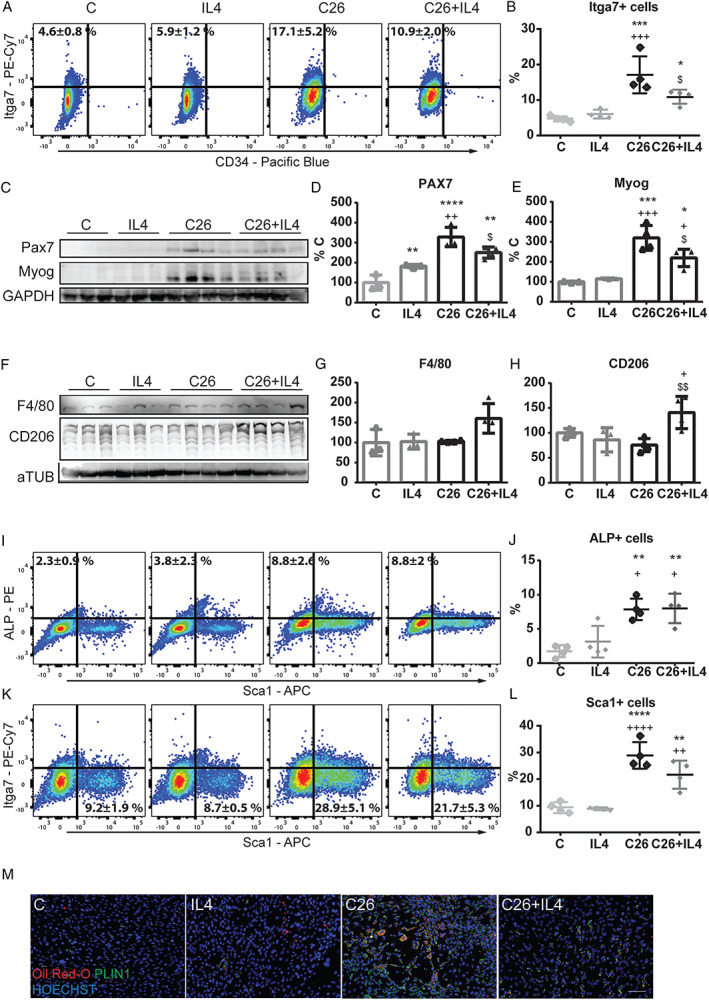
IL4 treatment counteracts muscle SC accumulation and decreases the levels of FAPs. After sacrifice and tissue digestion, muscle stem cells were analysed by flow cytometry for the expression of different markers of SCs (Itga7 and CD34) (*A*) and consequently (*B*, *D*, *F*) quantified. (*C*) The levels of PAX7 and myogenin (Myog) were analysed by WB, and (*D*, *E*) correspondent quantifications are reported on the right. (*F*) Protein levels of F4/80 and CD206 and (*G*, *H*) correspondent quantifications. (*I*) Flow cytometry analysis for the expression of markers for MABs (ALP); (*I*, *J*) quantification or for FAPs (Sca1) (*K*) and (*L*) quantification in C, IL4, C26, and C26 + IL4 muscles. (*M*) Populations of adipocyte‐like cells were stained with Oil‐Red‐O (red) and perilipin (PLIN1; green). Nuclei were stained with HOECHST (blue). Significance of the differences: ^*^ *P* < 0.05, ^**^ *P* < 0.01, ^***^ *P* < 0.001, ^****^ *P* < 0.0001 vs. C; ^+^ *P* < 0.05, ^++^ *P* < 0.01, ^+++^ *P* < 0.001, ^++++^ *P* < 0.0001 vs. IL4; ^$^ *P* < 0.05, ^$$^ *P* < 0.01 vs. C26.

To investigate if the beneficial effects induced by IL4 on muscle mass and performance could also result from improved myogenesis, we employed flow cytometry and established gating strategies to identify the myogenic stem cells present in the muscles of IL4‐treated and IL4‐untreated C26‐bearing mice. The total number of cells isolated from the muscle of IL4‐treated C26 hosts was comparable with controls and reduced with respect to untreated C26‐bearing mice (Supporting Information, *Figure S5*). As for the different muscle stem cell populations, the results showed that SC number was lower in C26 hosts administered with IL4 than in untreated tumour‐bearing animals (C26 = 17.1 ± 5.2%, C26 + IL4 = 10.9 ± 2.0%; *Figure* 5A and 5B). This reduction was paralleled by a significant decrease in PAX7 and myogenin protein levels (Myog; *Figure* 5C–5E) and by an increased number of type II macrophages (*Figure* 5F–5H). While no differences were appreciated for MABs (C26 = 8.8 ± 2.6%, C26 + IL4 = 8.8 ± 2.0%; *Figure* 5I and 5J), a trend to decrease, although not significant, was shown for FAPs (C26 = 28.9 ± 5.1%, C26 + IL4 = 21.7 ± 5.3%; *Figure* 5K and 5L). Similarly, immunofluorescence analysis for PDGFRa (known markers for FAP cells^13^) and Ki67 staining in C26 + IL4 muscle sections showed a lower accumulation of cells when compared with C26 ones (Supporting Information, *Figure S5b*). Consistently, the number of PLIN1 and Oil‐Red‐O‐positive cells was lower in primary cultures obtained from the muscle of IL4‐treated vs. IL4‐untreated C26‐bearing mice (*Figure* 5M), suggesting that interstitial FAPs in the former are less aberrant than in the latter.

### Interleukin‐4 administration affects skeletal muscle regeneration in C26‐bearing mice

**Figure 6:**
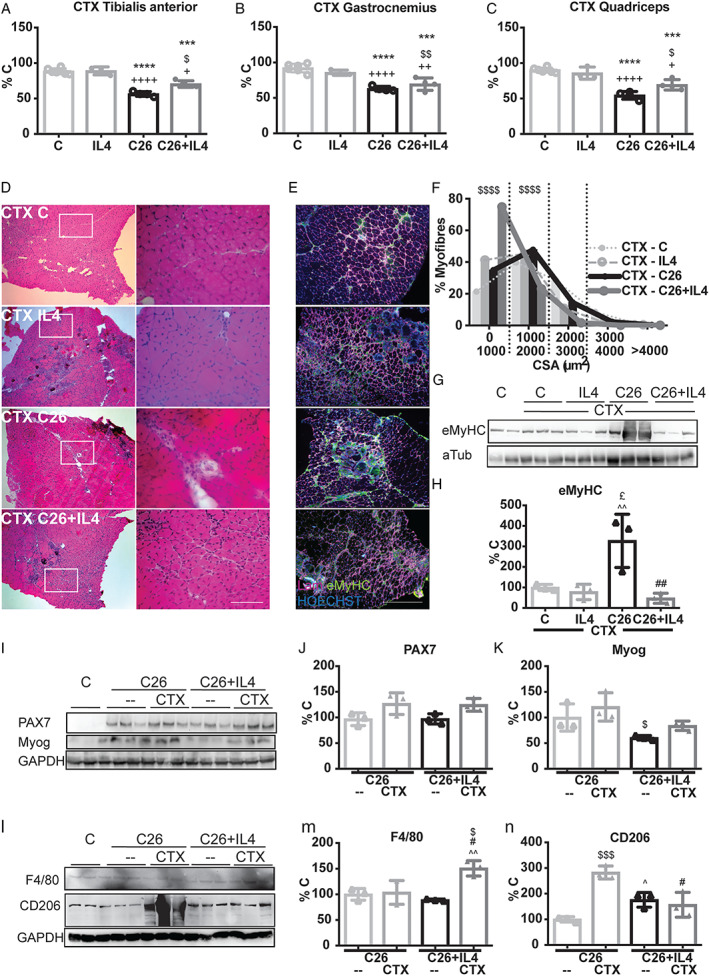
IL4 treatment induces a higher number of precursors to be activated after cardiotoxin (CTX)‐induced injury. In regeneration conditions, after 10 days from CTX‐induced muscle injury, (*A*) *TIB* (C: 97 ± 6 mg/100 g i.b.w.), (*B*) *GSN* (C: 262 ± 12 mg/100 g i.b.w.), and (*C*) *QD* (C: 369 ± 14 mg/100 g i.b.w.) muscle weights, expressed as percentage of the initial body weight. (*D*) H&E staining (column on the left) with insets from the different muscles (column on the right). (*E*) Representative IF muscle staining for Lam (purple) and eMyHC (green). Nuclei were stained with HOECHST (blue). (*F*) Fibre size distribution of *TIB* muscles from the different groups. (*G*) WB for eMyHC and (*H*) quantification. (*I*) PAX7, Myog protein levels with a representative Gapdh and their (*J*, *K*) quantifications. (*L*) Protein levels of F4/80 and CD206 in the same muscles have been as well quantified in (*M*) and (*N*). Significance of the differences is reported as ^***^ *P* < 0.001, ^****^ *P* < 0.0001 vs. C; ^++^ *P* < 0.01, ^++++^ *P* < 0.0001 vs. IL4; ^$^ *P* < 0.05, ^$$^ *P* < 0.01, ^$$$$^ *P* < 0.0001 vs. C26; ^£^ *P* < 0.05 vs. CTX IL4; ^#^ *P* < 0.05, ^##^ *P* < 0.01 vs. CTX C26; ^^^ *P* < 0.05, ^^^^ *P* < 0.01, ^^^^^ *P* < 0.001 vs. C26 + IL4.

Previous studies reported that muscle regeneration is delayed in tumour‐bearing mice.^7^, ^8^ On the other side, IL4 treatment was reported to improve muscle regeneration in glycerol‐injured animal model.^17^, ^27^ Along this line, the myogenic response following CTX‐induced muscle injury in the C26 hosts, in the presence or in the absence of IL4 treatment, was assessed. The results showed that regeneration is improved by treatment with the cytokine (*Figure* 6A–6C). H&E staining and quantification showed a decreased myofiber CSA in CTX‐C26 hosts and CTX‐IL4 mice compared with CTX‐C (*Figure* 6D and 6F; Supporting Information, *Figure S6a*). In CTX‐C26 hosts treated with IL4, myofibers were smaller than in the CTX‐C26‐bearing mice. This reduction in size, however, is counterbalanced by an increase in number of newly generated fibres with low CSA (diameter between 0 and 1000 μm^2^: C26 = 38%, C26 + IL4 = 78%; *Figure* 6D and 6F and Supporting Information, *Figure S6a*). Consistently, in muscles from CTX‐C26 hosts exposed to IL4, the levels of embryonic myosin heavy chain (eMyHC) were similar to CTX‐C or CTX‐IL4 muscles and lower than observed in muscles from CTX‐C26 hosts (*Figure* 6E, 6G, and 6H). Finally, in the injured muscle of C26 hosts treated with IL4 and analysed around 25–31 days after injury, the number of mature fibres characterized by peripheral nuclei was higher than in untreated injured C26‐bearing mice (that died 13 days after tumour transplantation and 8 days after CTX injury; Supporting Information, *Figure S6b*). No differences could be observed in both PAX7 and Myog expressions in both IL4‐treated and IL4‐untreated injured C26 hosts, although Myog levels showed a trend to decrease that did not reach significance (*Figure* 6I–6K). Finally, muscle type II macrophages were higher in CTX‐C26 hosts than in CTX‐C26‐bearing mice exposed to IL4 (*Figure* 6L–6N).

### Interleukin‐4 directly stimulates myocyte differentiation

**Figure 7:**
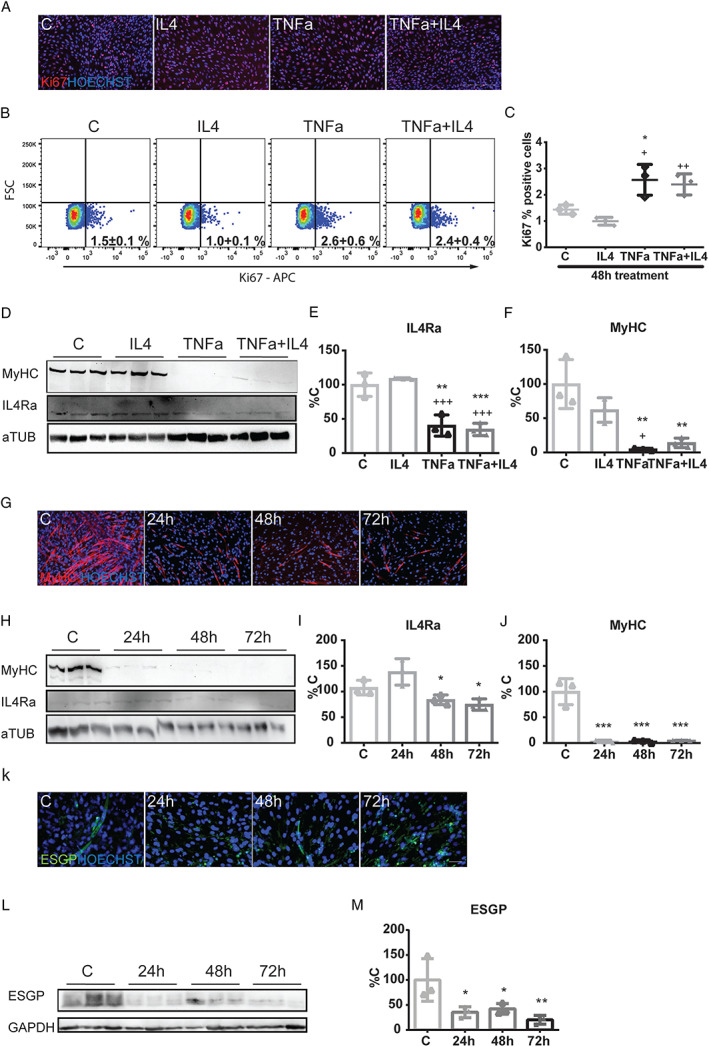
IL4 on C2C12 reduces the TNFα‐induced proliferative effect and is essential for myotube differentiation. (*A*) IF, (*B*) flow cytometry analysis, and (*C*) quantification of Ki67^+^ C2C12 cells treated with 10 ng/mL IL4, 100 ng/mL TNFα, or a combination of the two cytokines (TNFα + IL4) during 48 h of proliferating medium. (*D*) WB and (*E*) quantifications of the protein levels of MyHC and IL4R1a normalized for aTub after 10 ng/mL IL4 or 100 ng/mL TNFα or a combination of the two (TNFα + IL4) treatments during all C2C12 differentiation period (5 days differentiating medium). (*F*) IF for MyHC (red) on C2C12 during the three last days of differentiation (24 h = Day 3 of differentiation, 48 h = Day 4 of differentiation, 72 h = Day 5 of differentiation) after the double IL4Ra and IL13Ra silencing. HOECHST (blue) was used to stain nuclei. (*G*) WB and (*H*, *I*) quantifications for MyHC and IL4Ra protein levels normalized for aTub after double IL4Ra and IL13Ra silencing. (*J*) IF, (*K*) WB, and (*L*) quantification for the levels of ESGP (Myomerger) normalized on the ones of Gapdh that were analysed in the same cells silenced for IL4Ra and IL13Ra. Significance of the differences: ^*^ *P* < 0.05, ^**^ *P* < 0.01, ^***^ *P* < 0.001 vs. C; ^+^ *P* < 0.05, ^++^ *P* < 0.01, ^+++^ *P* < 0.001 vs. IL4.

To better understand the response of muscle stem cells to IL4, we first analysed the effects of IL4 on C2C12 myoblasts in proliferating conditions. Staining for Ki67 revealed an anti‐proliferative effect when 10 ng/mL IL4 were administered for 48 h to C2C12 myoblast cultures (*Figure* 7A). These results were confirmed by flow cytometry analysis and quantification (*Figure* 7B and 7C). In addition, IL4 was also able to reduce the stimulation of C2C12 myoblast proliferation induced by treatment with 100 ng/mL TNFα, a pro‐inflammatory cytokine known to inhibit C2C12 differentiation (*Figure* 7B and 7C). These observations suggested that IL4 could impact on C2C12 differentiation.^15^ To clarify this point, IL4/IL4Ra and IL13/IL13R mRNA levels were assessed at Days 0 and 5 of myogenic differentiation (Supporting Information, *Figure S7a*). As for protein, while IL4Ra levels were not detectable at Day 2 of differentiation (Supporting Information, *Figure S7b*), at Day 5, when completely differentiated myotubes were expressing MyHC, IL4Ra became detectable (*Figure* 7D–7F). When myotubes were exposed to 100 ng/mL TNFα during 5 days, a decrease in MyHC and in IL4Ra was reported. Such changes were not rescued by co‐treating myotubes with both TNFα and IL4. This pattern was maintained even if TNFα levels were reduced (50 ng/mL) and IL4 concentrations were increased (100 ng/mL IL4, Supporting Information, *Figure S7c* and *S7d*; 200 ng/mL IL4, Supporting Information, *Figure S7e* and *S7f*).

To better understand the role of the IL4‐dependent signalling in C2C12 differentiation, loss‐of‐function experiments were performed. IL4Ra was transiently silenced in differentiating C2C12. Interestingly, qRT–PCR data showed that the loss of IL4Ra can be compensated by IL13Ra. Indeed, when IL4Ra was down‐regulated, the levels of IL13Ra increased, likely compensating the missing IL4Ra (Supporting Information, *Figure S7g*). The other way around, when down‐regulating IL13Ra, IL4Ra levels increased (Supporting Information, *Figure S7h*), confirming our interpretation. IL4Ra silencing decreased transcript levels of genes promoting myoblast fusion,^28^, ^29^, ^30^ such as Myomixer (Mymx), Myomaker (Mymk), and Myomerger‐Minion (here indicated as Gm7325, analysed as short version and long version at the transcriptional level), to values lower than that of control cultures (Supporting Information, *Figure S7i*). Therefore, to obtain a more effective abolishment of IL4/IL13 signalling pathway, we induced a transient IL4Ra and IL13Ra esiRNA double down‐regulation in C2C12 induced to differentiate. The results confirmed that while in control C2C12 myotubes reached a complete differentiation, as shown by MyHC expression, transfected cells did not (*Figure* 7G–7J). Moreover, the protein levels of Myomerger (known also as embryonic stem cell and germ cell‐specific protein, ESGP) were reduced, confirming that the lack of IL4/IL13 signalling impaired myoblast fusion (*Figure* 7K–7M). Finally, WB analysis for ESGP protein levels in regenerating muscles from C26‐bearing mice did show similar levels in both IL4‐treated and IL4‐untreated CTX‐C26 hosts (Supporting Information, *Figure S7j* and *S7k*), suggesting that the delayed regenerative response observed in IL4‐treated vs. IL4‐untreated C26 hosts does not rely on impaired fusion.

## Discussion

The present study shows for the first time that cancer‐induced muscle wasting is associated with increased number of different muscle stem cell populations that cannot complete differentiation *in vivo* despite maintaining their intrinsic myogenic potential. Indeed, they are able to form mature myotubes when isolated from the cancer host micro‐environment. These results agree with previous data showing the accumulation of SCs in C26‐bearing mice.^7^, ^8^ The present results extend the analysis to MABs that, similarly to SCs, increase in number in the muscle of C26 hosts. These cells maintain the ability to fuse with existing myofibers when exposed to permissive regenerating conditions such as those occurring in the αSG‐null mice and are still able to respond to dorsomorphin.^11^ Taken together, these results highlight that stem cell number and their intrinsic myogenic differentiation ability are not the limiting factor contributing to muscle wasting in cachectic tumour‐bearing mice. In contrast, muscle interstitial stem cells seem to miss the input to differentiate. In this regard, myogenic differentiation is physiologically regulated by FAPs, a muscle stem cell population dependent on eosinophil granulocyte secretion of IL4 and IL13.^14^ When isolated from the muscle of the C26 hosts and put in culture, FAP cells degenerate to adipocyte‐like cells showing an adipogenic shift able to persist *in vitro* and probably suggesting an intrinsic modification of these cells. Modulations of muscle IL4/IL4R and IL13/IL13R, as well as of IL4 and IL13 plasma levels, showed that the signalling dependent on these cytokines is likely altered in the C26‐bearing mice, supporting the possibility of improper FAP behaviour and functions. In this regard, an intriguing hypothesis is that FAP degeneration to adipocyte‐like phenotype could contribute to myosteatosis, a common feature in patients affected by chronic inflammation and cachexia.^31^, ^32^ A detailed analysis of muscle biopsies obtained from cancer patients reported intramyocellular lipid droplets and large accumulation of neutral lipids in connective tissues in the *rectus abdominis*.^33^ Further studies are required to understand the nature of this phenomenon and to clarify if related to the excessive adipogenic differentiation of FAP cells that we report in C26 mice. In this regard, the lack of myosteatosis in the C26 hosts could be due to the short time interval between tumour implantation and animal sacrifice. Finally, altered FAP‐dependent signalling might also explain the impaired myogenesis occurring in tumour‐bearing mice (see succeeding text).

Along this line, the results of the present study show for the first time that IL4 administration to mice bearing the C26 tumour improves cachexia in terms of body weight, muscle mass, and function. Moreover, IL4‐treated C26‐bearing mice can survive 20 days longer than untreated C26 hosts. Similarly, increased survival was previously obtained by injecting intraperitoneally a plasmid encoding for IL4 in mice bearing an ascitic form of the C26 tumour.^34^ The effectiveness of IL4 administration in C26 hosts relies, partially at least, on the promotion of protein synthesis, as shown by puromycin incorporation and by sustained phosphorylation levels of Akt and p70. These kinases were shown to be unchanged, or even up‐regulated in the muscle of the C26 hosts,^4^, ^5^ likely due to an attempt to counteract the tumour‐induced hypercatabolic drift. In this regard, the data reported in the present study suggest that IL4 treatment is able to successfully push forward such compensatory response. The observation that no changes in both protein synthesis and anabolic kinase phosphorylation could be observed in IL4‐treated controls likely reflects the lack of effect in physiological states, where protein metabolism is not altered. Moreover, IL4 treatment results in decreased phosphorylation of some MAP kinases usually hyperactivated during cancer cachexia and shown to play a role in promoting muscle protein degradation.^4^, ^7^ This last observation is as well supported by lower levels of p62 accumulation in IL4‐treated than IL4‐untreated C26 hosts.

Interleukin‐4 administration to C26‐bearing mice also results in decreased SC number, while a trend towards reduction can be observed in FAPs. These latter, however, show a markedly reduced degeneration towards adipocyte‐like cells when isolated from the muscle of IL4‐treated C26 hosts and put in culture, supporting the idea that a restored IL4/IL13 signalling can decrease the degeneration of muscle stem cells in C26‐bearing mice. These findings are in accordance with the reduced muscle atrophy and fibrosis obtained in *mdx* mice by preventing FAP degeneration.^15^, ^35^ The delay in muscle regeneration occurring in the C26 hosts is partially corrected by IL4 treatment, as shown by the increased number of myofibers with central nuclei, that slowly reach the mature phenotype, characterized by low expression of eMYHC. Interestingly, these fibres are not blocked in their growth, but they rather undergo a temporary delay, showing, after a longer time period, several fully matured fibres with peripheral nuclei. In line with our results, a recent study showed that IL4‐conjugated gold nanoparticles injected into injured murine skeletal muscle improved muscle histology and performance associated with two‐fold increase of type II macrophages.^36^

The idea that IL4 treatment in the C26 hosts acts on the muscle by stimulating myogenic differentiation, likely through improved FAP activity, is supported by the results obtained on C2C12 cultures exposed to the cytokine. Indeed, IL4 not only is able to inhibit myoblast proliferation prompting them to differentiation but also counteracts, partially at least, the inhibition of differentiation induced by TNFα. In this regard, TNFα maintains C2C12 cultures in a proliferative setting,^37^ which could partially explain the accumulation of SCs observed in the muscle of tumour‐bearing mice. Interestingly, the inhibition of C2C12 differentiation by TNFα is associated with reduced levels of IL4Ra. Thus, increasing IL4 concentrations do not rescue MyHC levels. The ability of IL4 to guide myogenesis is in line with previous data obtained in human muscle cells.^16^, ^17^ The involvement of IL4 in myogenesis is further supported by the observation that loss of function of both IL4Ra and IL13Ra during differentiation impairs the ability of myoblasts to fuse. As for the mechanisms by which the lack of IL4‐dependent signalling may inhibit differentiation, down‐regulation of Myomerger (ESGP) protein levels as well as of other genes relevant to fusion has been shown to occur. These results demonstrate that physiologic expression of IL4Ra is necessary to determine myocyte fusion, in C2C12 cultures at least, and it is not dispensable to complete myotube differentiation.

A possible limitation in the present study is that increased tumour mass occurs when IL4 is administered to C26‐bearing mice. In this regard, in colorectal cancer stem cells, protection from apoptosis is achieved by autocrine production of IL4 through up‐regulation of anti‐apoptotic mediators.^38^ These observations could introduce a note of care to the possibility that IL4 treatment can be proposed as a strategy to counteract cancer cachexia. However, despite being increased in size, tumours at Day 31 in IL4‐treated C26 hosts present with conspicuous necrotic areas and with a high number of infiltrating inflammatory/immune cells (CD8^+^ lymphocytes and type II macrophages), which could be involved in tumour cytotoxicity and necrosis. These results suggest that IL4 administration to C26‐bearing mice, far from stimulating cancer growth, increases the immune response against the tumour. However, this observation is in apparent contrast with the well‐known anti‐inflammatory action of IL4. In this regard, further experiments are required to clarify if systemic IL4 administration might exert different actions at the tumour and muscle levels, respectively. Consistently, the observations here reported show that *in vitro* IL4 is able to modulate myogenesis but not to affect C26 tumour cell growth curve, suggesting that while the effects on muscle are directly mediated by IL4, those on the tumour could depend on additional mediators.

In conclusion, these results demonstrate the importance of IL4 as a cytokine able to impinge on the inflammatory system, promoting an anti‐inflammatory muscle micro‐environment, and to raise the state of activation of anabolic kinases involved in the maintenance of muscle protein synthesis. Of interest, IL4 shares at least some beneficial actions with exercise, previously shown to reduce systemic inflammation^39^ and to increase muscle protein synthesis.^21^ In this regard, the hypothesis that IL4 could work as a sort of exercise‐mimetic drug could be proposed, although further analyses aimed at assessing muscle energy metabolism after IL4 administration are required to clarify this point. On the whole, the results shown in the present study highlight that IL4/IL13 signalling is effective in keeping the correct equilibrium between anabolic and catabolic cues. Physiologic levels of IL4/IL13 are probably responsible for the normal homeostasis of muscle tissue; when dysregulated, they can contribute to muscle atrophy. Further efforts should be performed to determine if the administration of IL4 could ameliorate cancer‐induced muscle atrophy when associated to chemotherapeutic drugs and perhaps extend this treatment to other disorders where muscle precursor accumulation occurs, such as muscular dystrophies (at least in the initial phases of the disease) or myotonic dystrophies, also characterized by chronic inflammation.

## Author contributions

D.C. participated in conception and design, collection and assembly of data, data analysis and interpretation, and manuscript writing; R.D. participated in flow cytometry collection and assembly of data, data analysis and interpretation; F.P. participated in data interpretation; D.N. participated in data interpretation; P.C. participated in conception and design, data analysis and interpretation, manuscript writing, and final approval of the manuscript; M.S. participated in conception and design, data analysis and interpretation, manuscript writing, and final approval of the manuscript.

## Conflict of interest

None declared.

## Supporting information

Figure S1: Characterization of C‐ and C26‐SCs and MABs. a) IF of SCs from C and C26 muscles stained for ACTN2 (red) and MyoD (green; at day 2 of differentiation). Nuclei were stained with HOECHST (blue). Scale bar: 500 μm. WB for MyHC and aTub on b) GFP^+^ C‐ and c) GFP^+^ C26‐MABs in co‐culture with C2C12 at day 0 and 5 of myotube differentiation. d) qRT‐PCR for the expression of IL4, IL4Ra1, IL13 and IL13R in C and C26 muscles normalized for the housekeeping genes Gapdh, HPRT, TBP. Significance of the differences: *p < 0.05, ****p < 0.0001 vs C.
Figure S2: IL4 treatment. a) C26 number of cells treated for 72 h *in vitro* with 10, 100 and 500 ng/ ml IL4. b) Absorption curve of IL4 in Balb/c mice at different time points after 1.3 ug IL4 administration (n = 3 per time point). c) Tumor weight in C26 and C26 + IL4 at the day of the sacrifice (14 days after tumor cell injection; n = 4 per group). d) Quadriceps muscle weight of C, IL4, C26 and C26 + IL4 mice (C: 410 ± 33 mg/100 g i. b. w.). Representative flow cytometry analysis on cells extracted after peritoneal lavage from (e, h) 1 mg/kg LPS injected mice (as positive control), or C26 and C26 + IL4 peritoneal lavage was analyzed for the main markers of (f) lymphocytes and (g) quantified or for (i) macrophage populations and (j) quantified. Significance of the differences **p < 0.01, ***p < 0.001, ****p < 0.0001 vs C; ++p < 0.01, +++p < 0.001 vs IL4; $p < 0.05, $$$p < 0.001 vs C26.
Figure S3: Flow cytometry analysis of circulating immune cells. Representative flow cytometry analysis of the circulating immune cells from C26 and C26 + IL4 mice for the main markers and quantifications of (a) lymphocytes and (b) macrophages. Significance of the differences $$$p < 0.001 vs C26.
Figure S4: analysis of other important protein expressions in muscle tissue. a) WB for p38 MAP‐Kinase and (b) quantification. c) WB for nuclear extract of P‐STAT3 and P‐STAT6 with (d, e) respective quantifications. f) WB for Atrogin1 and TRIM32 with (g, h) respective quantification. i) WB for Beclin1, LC3B, p62 and (j ‐ l) respective quantifications. m) WB for the comparison of p62 levels between C26 and C26 + IL4 and (n) quantification. All the values were normalized for the total protein levels and on aTub or GAPDH expression levels. Significance of the differences is reported as *p < 0.05, **p < 0.01, ***p < 0.001 vs C; ++p < 0.01, +++p < 0.001 vs IL4; $p < 0.05 vs C26.
Figure S5: Numbers of cells extracted from muscle of C, IL4, C26 and C26 + IL4 normalized for the muscle mass. a) Cells were counted immediately after tissue digestion. b) IF on muscle slides for the expression of PDGFRa (green) and Ki67 (magenta) from C, IL4, C26, C26 + IL4 muscles. HOECHST (blue) was used to stain nuclei. The arrows indicate double positive nuclei surrounded by a green signal. *p < 0.05 vs C; ++p < 0.01 vs IL4; $$$ p < 0.001 vs C26. Scale bar: 100 μm.
Figure S6: IL4‐treated muscles do not show impairment in regeneration after CTX injury. a) Average of fibre CSA in muscles after 10 days of CTX injection. b) H&E staining of muscle sections taken at longer time points after CTX injury was induced. In particular, for CTX‐C26 (n = 5), 9 to 12 days after CTX injury, and for CTX‐C26 + IL4 mice (n = 5), 16 to 30 days after CTX injury. Significance of the differences ***p < 0.001 vs C; +++p < 0.001 vs IL4; $$$p < 0.001 vs C26.
Figure S7: Increasing concentrations of IL4 dos not rescue 50 ng/ ml TNFa‐impaired myotube differentiation. a) qRT‐PCR for the expression of IL4, IL4Ra, IL13 and IL13R in C2C12 at day 0 and 5 of myotube differentiation normalized for the housekeeping genes Gapdh, HPRT, TBP. b) C2C12 myoblasts at day 2 of differentiation were analyzed for IL4R expression on the levels of aTUB. c) MyHC levels by WB were normalized for aTub and quantified in C2C12 treated with low concentration of TNFa (50 ng/ml) and (d) 100 ng/ ml IL4 or (e‐f) 200 ng/ml IL4. g) Silencing for IL4R1a and (h) for IL13R1a in myotubes was measured by qRT‐PCR during the three last days of differentiation (esi24h = day 3, esi48h = day 4, esi72h = day 5 of differentiation medium). i) Levels of Myomixer (Mymx), Myomaker (Mymk) and Myomerger (Gm7325s and Gm7325l) were analyzed on the IL4R1a silenced cells by qRT‐PCR normalized for the housekeeping genes Gapdh, HPRT, TBP. j) WB and (k) quantification for ESGP (Myomerger) on the levels of GAPDH in CTX‐C CTX‐IL4, CTX‐C26 and CTX‐C26 + IL4 muscle protein extract. Significance of the differences *p < 0.05, **p < 0.01, ***p < 0.001, ****p < 0.0001 vs C; +p < 0.05, +++p < 0.001 vs IL4. £p < 0.05 vs CTX‐C; ^p < 0.05 vs CTX‐IL4.
