# Supplementary figures and images for: Interleukin‐4 administration improves muscle function, adult myogenesis, and lifespan of colon carcinoma‐bearing mice
Source: J Cachexia Sarcopenia Muscle. 2020 Feb 27;11(3):783–801. doi: 10.1002/jcsm.12539 (PMC7296260; doi:10.1002/jcsm.12539)

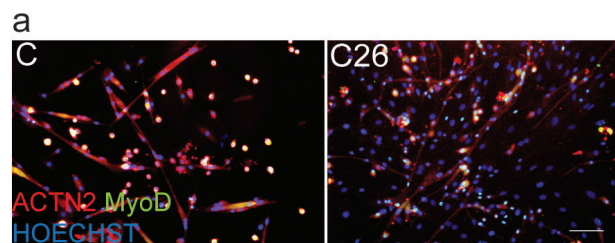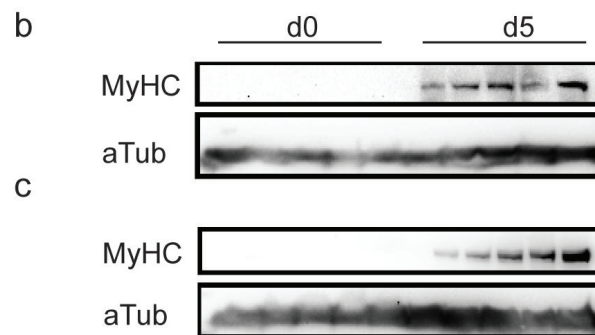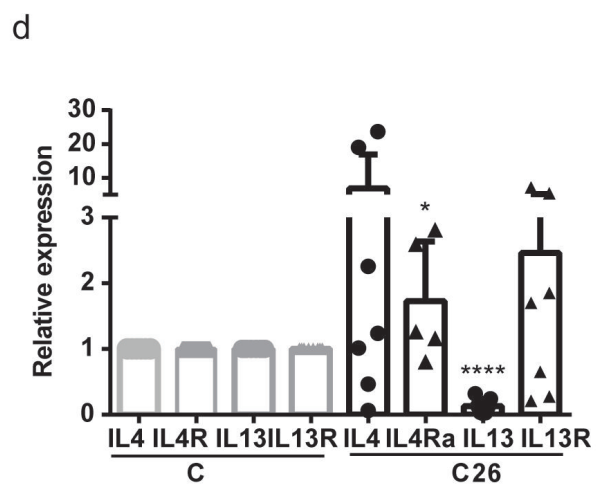

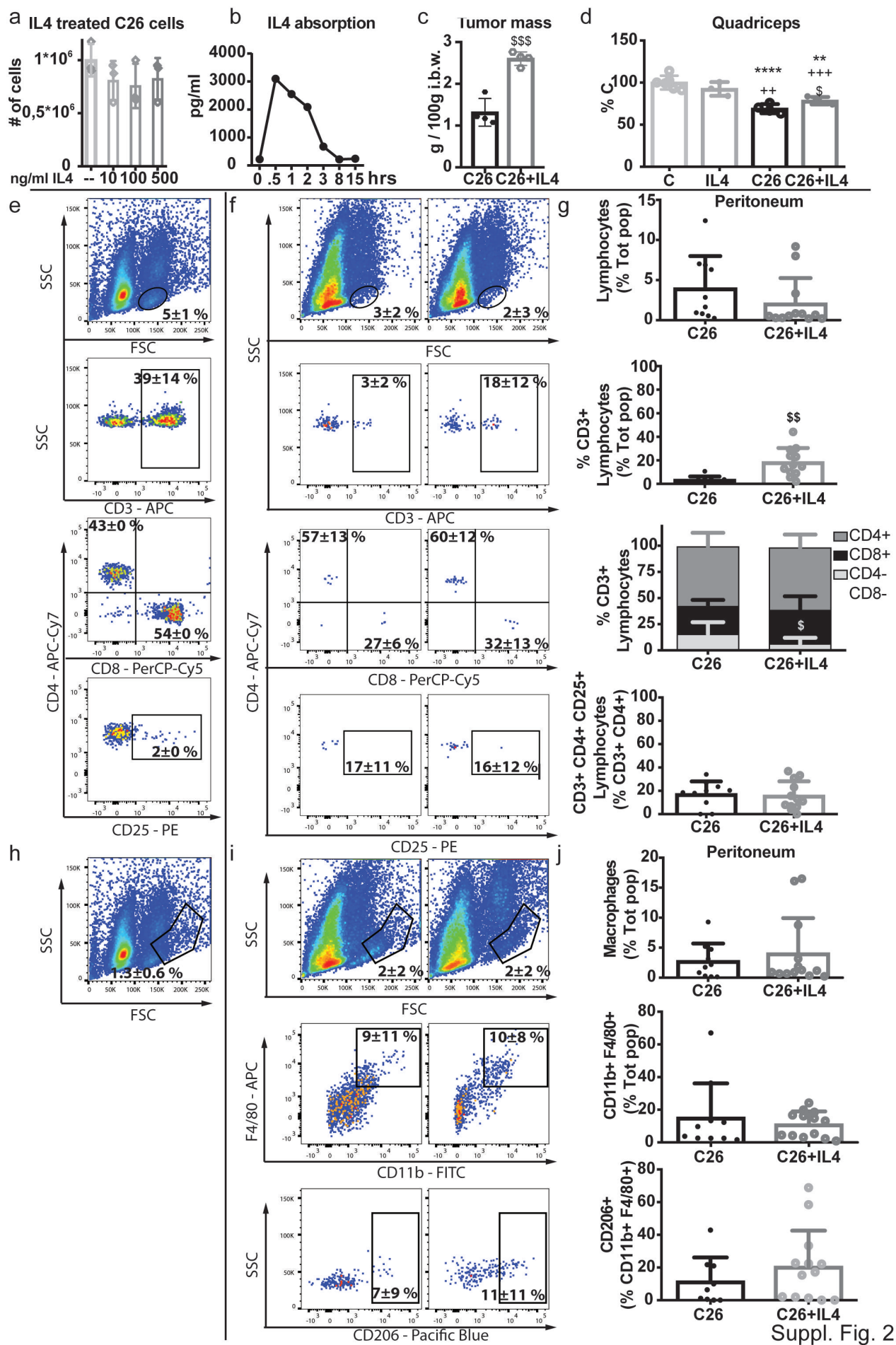

Suppl. Fig. 2

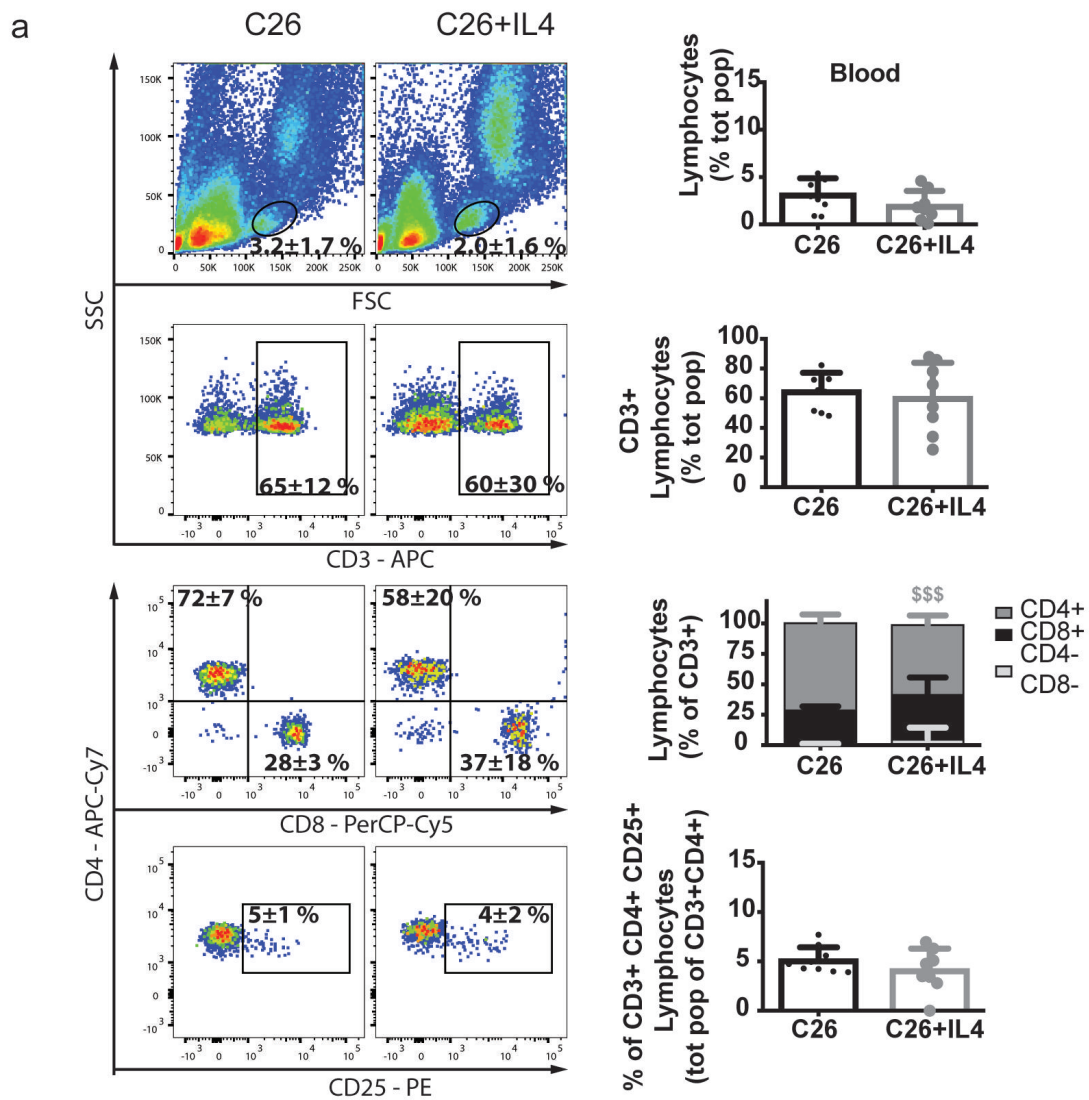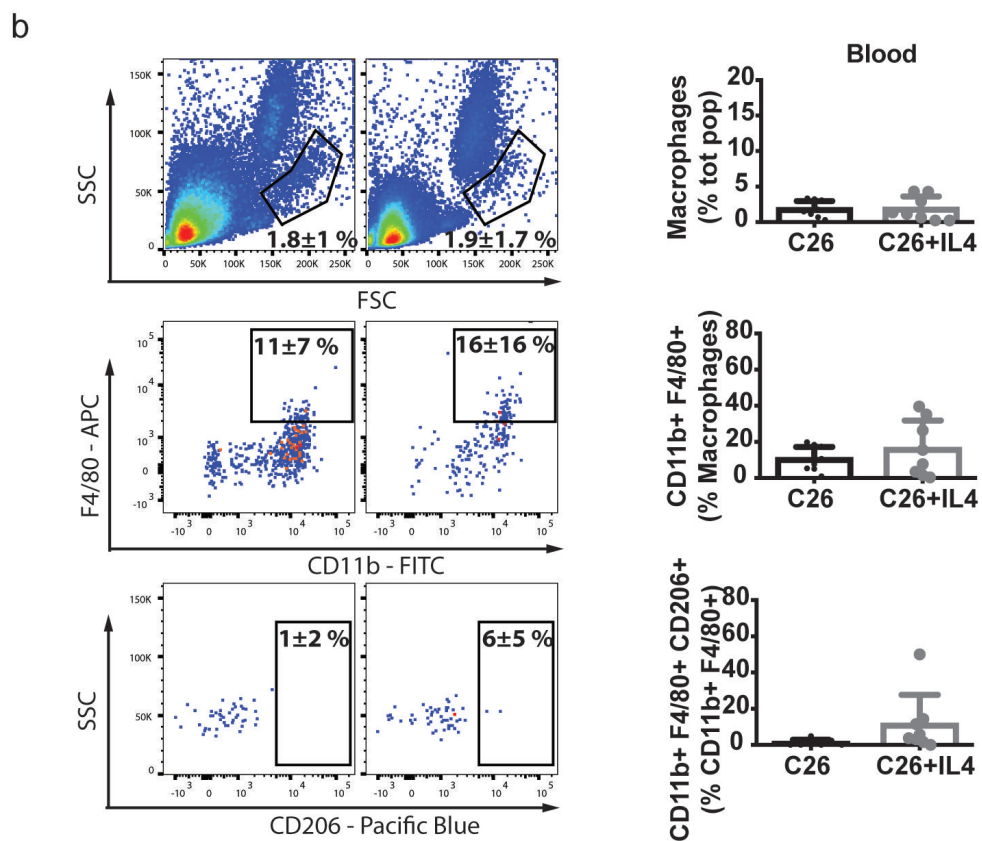

Suppl. Fig. 3

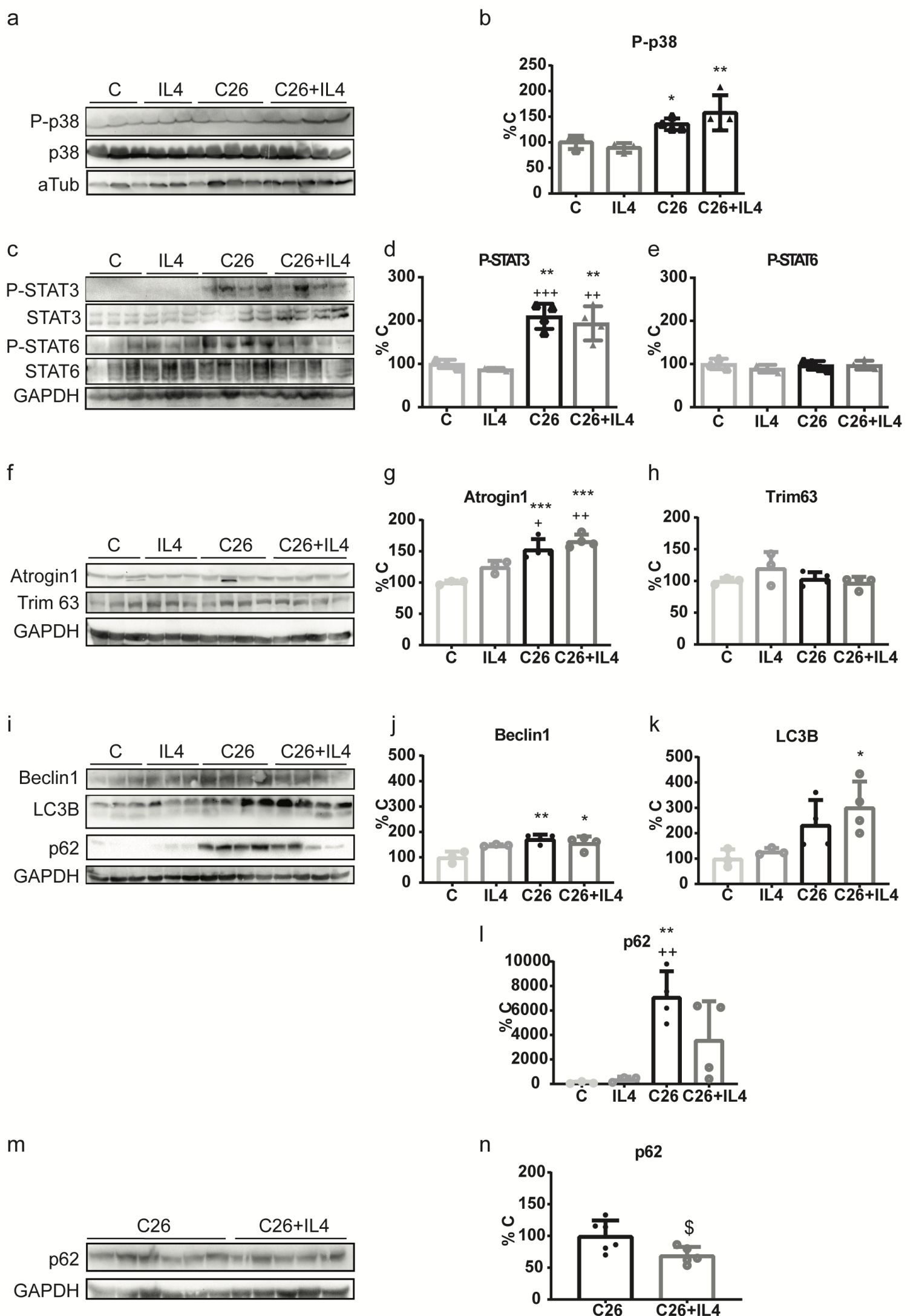

a

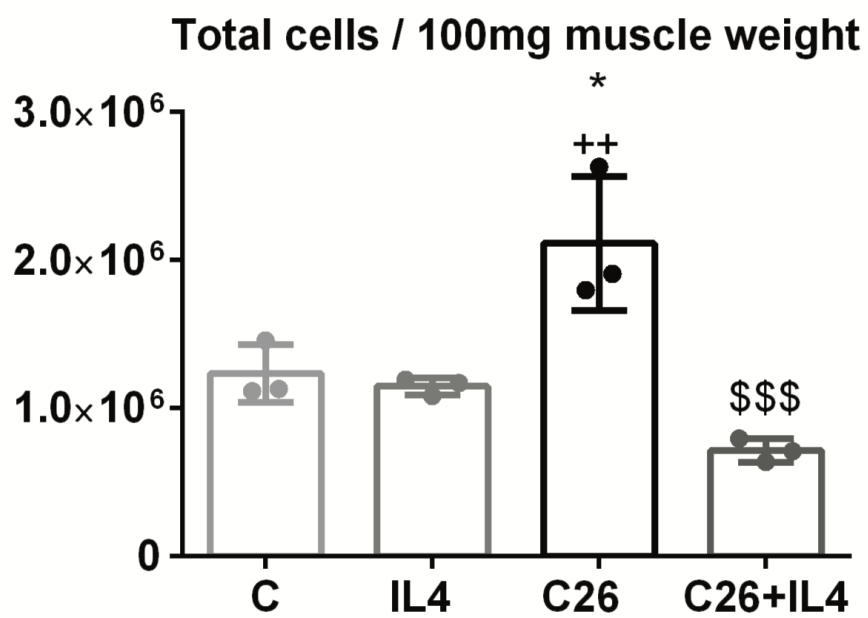

b

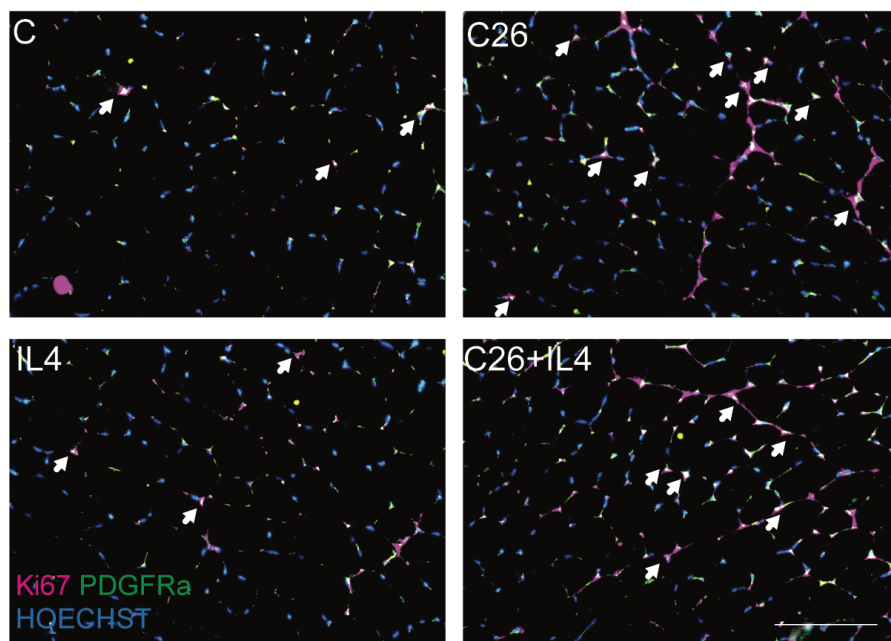

a

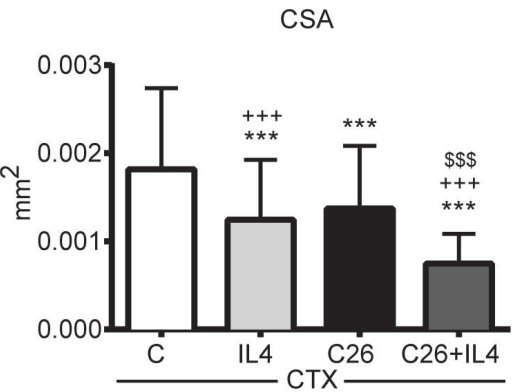

b

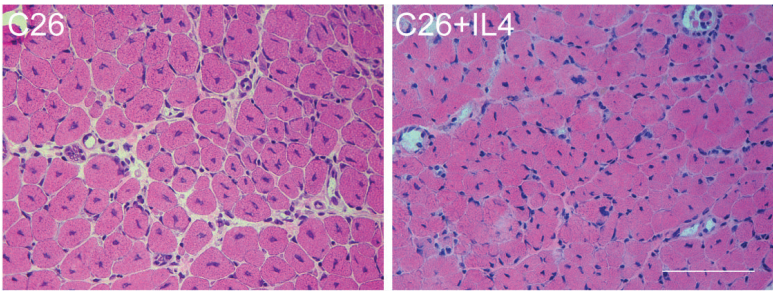

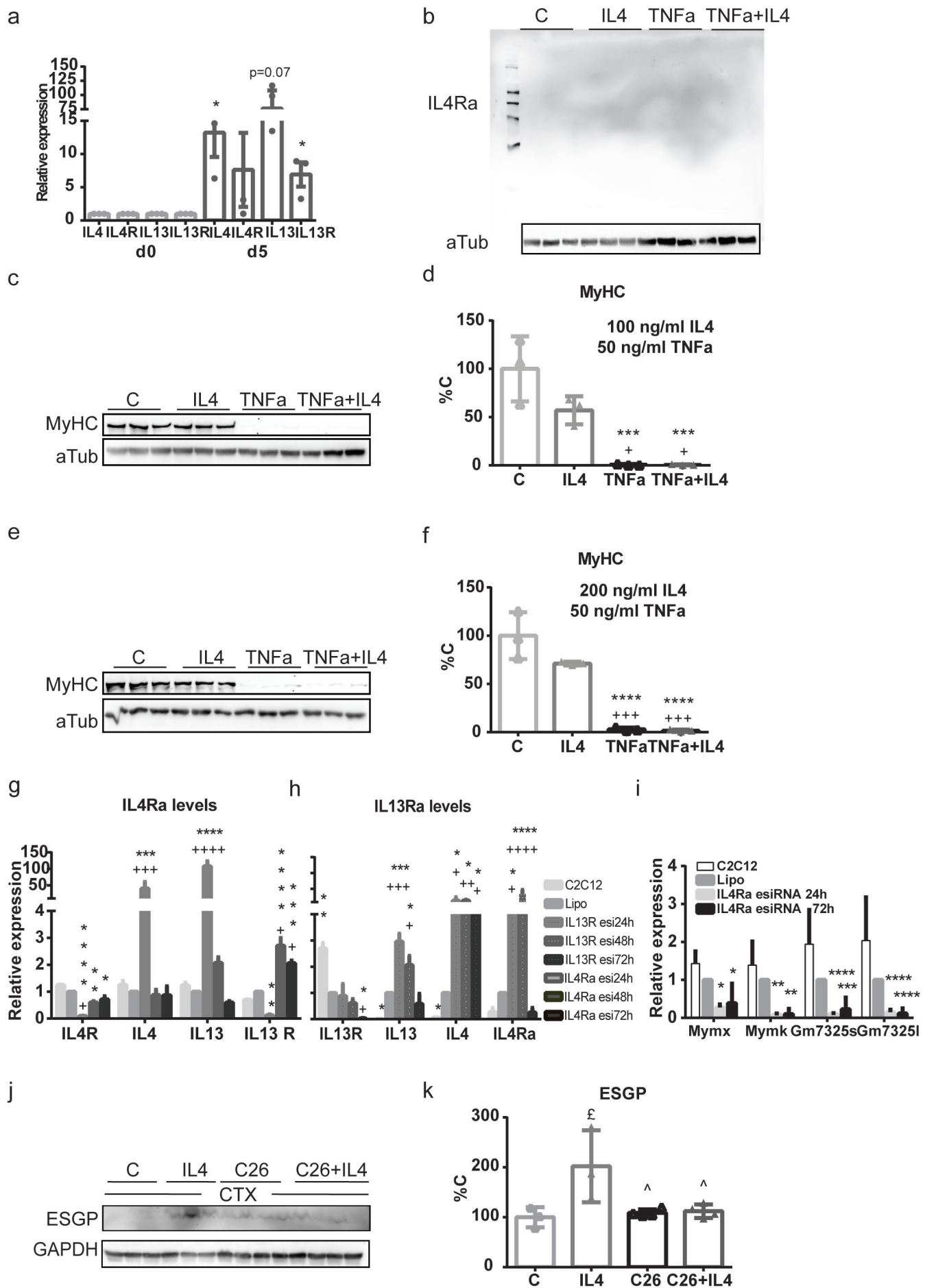

Suppl. Fig. 7

Supplement: Supplementary file 1 — Figure S1: Characterization of C‐ and C26‐SCs and MABs. a) IF of SCs from C and C26 muscles stained for ACTN2 (red) and MyoD (green; at day 2 of differentiation). Nuclei were stained with HOECHST (blue). Scale bar: 500 μm. WB for MyHC and aTub on b) GFP+ C‐ and c) GFP+ C26‐MABs in co‐culture with C2C12 at day 0 and 5 of myotube differentiation. d) qRT‐PCR for the expression of IL4, IL4Ra1, IL13 and IL13R in C and C26 muscles normalized for the housekeeping genes Gapdh, HPRT, TBP. Significance of the differences: *p < 0.05, ****p < 0.0001 vs C. Figure S2: IL4 treatment. a) C26 number of cells treated for 72 h in vitro with 10, 100 and 500 ng/ ml IL4. b) Absorption curve of IL4 in Balb/c mice at different time points after 1.3 ug IL4 administration (n = 3 per time point). c) Tumor weight in C26 and C26 + IL4 at the day of the sacrifice (14 days after tumor cell injection; n = 4 per group). d) Quadriceps muscle weight of C, IL4, C26 and C26 + IL4 mice (C: 410 ± 33 mg/100 g i. b. w.). Representative flow cytometry analysis on cells extracted after peritoneal lavage from (e, h) 1 mg/kg LPS injected mice (as positive control), or C26 and C26 + IL4 peritoneal lavage was analyzed for the main markers of (f) lymphocytes and (g) quantified or for (i) macrophage populations and (j) quantified. Significance of the differences **p < 0.01, ***p < 0.001, ****p < 0.0001 vs C; ++p < 0.01, +++p < 0.001 vs IL4; $p < 0.05, $$$p < 0.001 vs C26. Figure S3: Flow cytometry analysis of circulating immune cells. Representative flow cytometry analysis of the circulating immune cells from C26 and C26 + IL4 mice for the main markers and quantifications of (a) lymphocytes and (b) macrophages. Significance of the differences $$$p < 0.001 vs C26. Figure S4: analysis of other important protein expressions in muscle tissue. a) WB for p38 MAP‐Kinase and (b) quantification. c) WB for nuclear extract of P‐STAT3 and P‐STAT6 with (d, e) respective quantifications. f) WB for Atrogin1 and TRIM32 with (g, h [file JCSM-11-783-s001.pdf]
